# Microstructural Insights Into LATP Ceramic Nanofibers for High‐Performance Quasi‐Solid‐State Batteries

**DOI:** 10.1002/advs.202510846

**Published:** 2025-11-20

**Authors:** Sivaraj Pazhaniswamy, Matteo Bianchini, Shweta Hiwase, Seema Agarwal

**Affiliations:** ^1^ Department of Materials University of Oxford Parks Road Oxford OX1 3PH United Kingdom; ^2^ Bavarian Center of Battery Technology (BayBatt) Weiherstraße 26 95448 Bayreuth Germany; ^3^ Faculty of Biology, Chemistry and Earth Sciences University of Bayreuth Universitätstraße 30 95447 Bayreuth Germany; ^4^ Advanced Sustainable Polymers Macromolecular Chemistry II University of Bayreuth 95440 Bayreuth Germany

**Keywords:** ceramic nanofiber, dendrite suppression, electrospinning, polymer–ceramic composite, solid‐state metal battery

## Abstract

Composite solid‐state electrolytes (CPEs) offer great potential for advancing quasi‐solid‐state lithium metal batteries (QSLMBs) due to their high ionic conductivity, electrochemical performance, and thermal stability. However, conventional CPEs, formed by incorporating ceramic particles into polymer matrices, often fail to significantly improve critical current density and rate performance. This study presents a green synthesis of NASICON‐type Li_1_._4_Al_0_._4_Ti_1_._6_(PO_4_)_3_ ceramic nanofibers (LATP‐NFs) via electrospinning. It optimizes parameters such as solvent type, polymer and LATP precursor concentrations, heating rates, and calcination temperatures to control LATP‐NF microstructures. Embedding 30 wt.% LATP‐NF (LATP‐30) into a poly(vinylidene fluoride)‐lithium bis(trifluoromethanesulfonyl)imide (PVDF‐LiTFSI) matrix yields a CPE with reasonable ionic conductivity of 0.21 mS cm^−1^ at room temperature (RT), good thermal and electrochemical stability (>5 V), and enhanced mechanical strength. LATP‐30 effectively suppresses lithium dendrite growth, achieving a high critical current density of 10 mA cm^−2^. The LFP|LATP‐30|Li cell delivers 169 mAh g^−1^ at 0.1 C and maintains capacities of 122, 111, and 101 mAh g^−1^ at 3, 5, and 10 C, respectively. It retains 153 mAh g^−1^ after 300 cycles, with 97% capacity retention at 0.5C. This work demonstrates a sustainable and non‐toxic strategy for synthesizing LATP‐NFs for high‐performance QSLMBs.

## Graphical abstract

A sustainable electrospinning technique produces LATP ceramic nanofibers that form continuous Li⁺‐conductive pathways within composite solid electrolytes. The resulting quasi‐solid‐state batteries combine the strength of ceramic nanofibers with the flexibility of polymers, delivering high critical current density and excellent rate performance at room temperature—bridging the gap between liquid and solid‐state lithium battery technologies.

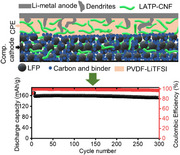

## 1 Introduction

All‐solid‐state batteries (SSBs) are considered to be the most secure, adaptable, high–energy‐density, and long‐lasting energy storage devices for electric vehicles and grid energy storage applications. However, poor solid–solid interfacial contact, low ionic conductivity at RT, especially ceramic/inorganic solidstate electrolytes (SSEs) are brittle, prone to cracking under stress, leading to interface degradation or failure. Solid‐state interfaces generally require high‐pressure or high‐temperature processing to achieve strong adhesion and optimal electrochemical performance. This complicates manufacturing, increases costs, and hinders practical implementation. These limitations have driven growing interest in quasi‐solid‐state lithium metal batteries. The QSLMBs are electrochemical cells that utilize SSEs or CPEs containing a small fraction of liquid electrolyte (<10%). These electrolytes exhibit mechanical stability and safety similar to solid‐state systems while maintaining ionic conductivity comparable to liquid electrolytes. In such systems, the minor liquid phase enhances Li⁺ coordination and interfacial wettability, thereby forming continuous Li⁺ migration channels across the polymer–ceramic–electrode interfaces. This hybrid ion transport mechanism improves Li⁺ mobility and interfacial contact without compromising the solid‐like integrity of the electrolyte. QSLMBs thus represent a significant advancement in energy storage technology and hold strong potential for practical applications. This paradigm shift offers numerous advantages, including substantially lower interfacial impedance and enhanced safety by reducing the liquid component by ≈90%, thereby minimizing the risk of fires and explosions compared to conventional lithium‐ion batteries (LIBs).^[^ ^1^, ^2^ ^]^ Furthermore, QSLMBs attain enhanced energy density through the use of lithium metal anodes, allowing for compact and lightweight configurations that are particularly advantageous for electric vehicles and portable electronic devices.^[^ ^3^, ^4^ ^]^ Despite these benefits, challenges remain in achieving efficient ionic conductivity at RT and ensuring long‐term interfacial stability and compatibility between electrolytes and electrodes.^[^ ^5^ ^]^ Nonetheless, continued research and development in QSLMB technology promise to revolutionize energy storage with safer, more efficient, and higher‐performance batteries.

SSE encompasses ceramics, polymers, and composite materials. Ceramic electrolytes such as NASICON‐type LATP (Li_1_₊_x_Al_x_Ti_2_₋_x_(PO_4_)_3_), lithium garnets (Li_7_La_3_Zr_2_O_12_), and inorganic sulfides (e.g., Li_10_GeP_2_S_12_, Li_6_PS_5_Cl) are well known for their high ionic conductivity and stability.^[^ ^6^, ^7^, ^8^, ^9^, ^10^ ^]^ Among these, LATP stands out due to its superior air stability, ionic conductivity, and structural robustness. Partial substitution of aluminum (Al) for titanium (Ti) in the LATP lattice increases lithium‐ion concentration and enhances conductivity. LATP exhibits ionic conductivities in the range of 0.1–1 mS cm^−1^ at RT and features simple synthesis routes using commercially feasible raw materials.^[^ ^9^ ^]^ However, practical implementation is limited by high sintering temperatures, brittleness, and the formation of resistive interfaces between the electrolyte and electrodes. Polymer electrolytes offer flexibility and ease of processing but are generally constrained by lower ionic conductivity, narrow electrochemical stability windows, and the need for elevated operating temperatures. CPEs bridge these gaps by incorporating ceramic particles into polymer matrices, thereby enhancing ionic conductivity while maintaining mechanical flexibility and facile fabrication.^[^ ^11^, ^12^, ^13^ ^]^

Numerous studies have reported CPE fabrication through the incorporation of active ceramic nano/microparticles (e.g., LLZO, LATP, LLTO) and inactive fillers (e.g., TiO_2_, SiO_2_, Al_2_O_3_).^[^ ^14^, ^15^, ^16^ ^]^ However, ceramic particles tend to agglomerate, resulting in non‐uniform dispersion and discontinuous ion transport pathways within the CPE.^[^ ^16^ ^]^ Recently, the incorporation of ceramic nanofibers into polymer matrices has emerged as a promising solution, significantly enhancing ionic conductivity, mechanical strength, and dendrite suppression. The unique 3D interconnected structure and high surface area‐to‐volume ratio of ceramic nanofibers facilitate continuous ion transport pathways within the polymer matrix.^[^ ^5^, ^17^, ^18^, ^19^, ^20^ ^]^ These attributes make ceramic nanofibers an attractive choice for advanced QSLMBs. However, only a limited number of studies have explored LATP‐NFs and their electrochemical performance.^[^ ^21^, ^22^, ^23^ ^]^ LATP‐NFs are typically fabricated via electrospinning, followed by thermal treatment to crystallize the LATP phase and form the desired nanofibrous structure. This process yields nanofibers with diameters ranging from tens to hundreds of nanometers, forming high‐surface‐area networks. For example, La Monaca et al.^[^ ^21^ ^]^ synthesized LATP‐NFs using N,N‐Dimethylformamide‐Tetrahydrofuran (DMF–THF) as solvents for the electrospinning precursor solution and reported an ionic conductivity of 3 × 10^−^⁵ S cm^−1^ at RT. The same group prepared NASICON‐type Li_1_._5_Al_0_._5_Ge_1_._5_₋_x_Ti_x_(PO_4_)_3_ (LAGTP) ceramic nanofibers and investigated their electrochemical performance in Poly(ethylene oxide) (PEO)–LAGTP‐based solid electrolytes.^[^ ^22^ ^]^ Similarly, Yu et al.^[^ ^23^ ^]^ fabricated LATP‐NFs and developed hybrid solid electrolytes (HSE) incorporating single‐ion‐conducting polymers. These HSEs exhibit stable lithium stripping/plating performance at 0.1 mA cm^−2^ and rate capability up to 1 C in LFP|HSE|Li cells. Despite the advantages of LATP‐NFs, their development faces challenges such as the use of hazardous solvents (e.g., DMF), which complicate manufacturing and raise environmental concerns. Moreover, no studies have systematically explored the synthesis of LATP‐NFs using non‐toxic, green solvents (e.g., water, ethanol) or examined the influence of parameters such as solvent type, heating rate, and calcination temperature on their microstructure.

The present work focuses on the fabrication of LATP‐NFs via electrospinning using environmentally friendly solvents and two different polymer host materials. To the best of our knowledge, this is the first comprehensive investigation into the effects of these parameters on the microstructural properties of LATP‐NFs and their influence on electrochemical performance in CPEs. The CPEs are fabricated by incorporating various LATP‐NF structures into PVDF–LiTFSI matrices and evaluated in QSLMBs. The integration of LATP‐NF enhances the polymer‐ceramic interface and plasticizes PVDF by lowering its crystallinity, creating amorphous regions that improve lithium‐ion conduction. In the CPEs, Li⁺ ions coordinate primarily with the polar functional groups of the polymer and the oxygen atoms in the LATP framework, while the small amount of liquid electrolyte facilitates dynamic Li⁺ transport pathways at the polymer–ceramic interface. As a result, the incorporation of 6 µL cm^−2^ of liquid electrolyte (1 M LiPF_6_ in EC:DMC = 1:1 vol% with 5% FEC) at both the Li–CPE and CPE–LFP interfaces enables stable operation at a high critical current density (CCD) of 10 mA cm^−2^ and delivers enhanced rate capability up to 10 C. Post‐cycling Electrochemical Impedance Spectroscopy (EIS) and Field‐Emission Scanning Electron Microscopy (FE‐SEM) analyses confirm that LATP‐NF effectively suppresses dendrite penetration. This study demonstrates the potential of green synthesis methods for LATP‐NFs to reduce environmental impact, simplify production, and lower manufacturing costs.

## 2 Results and Discussion

### 2.1 Microstructural Properties of LATP Ceramic Nanofibers

**Figure 1:**
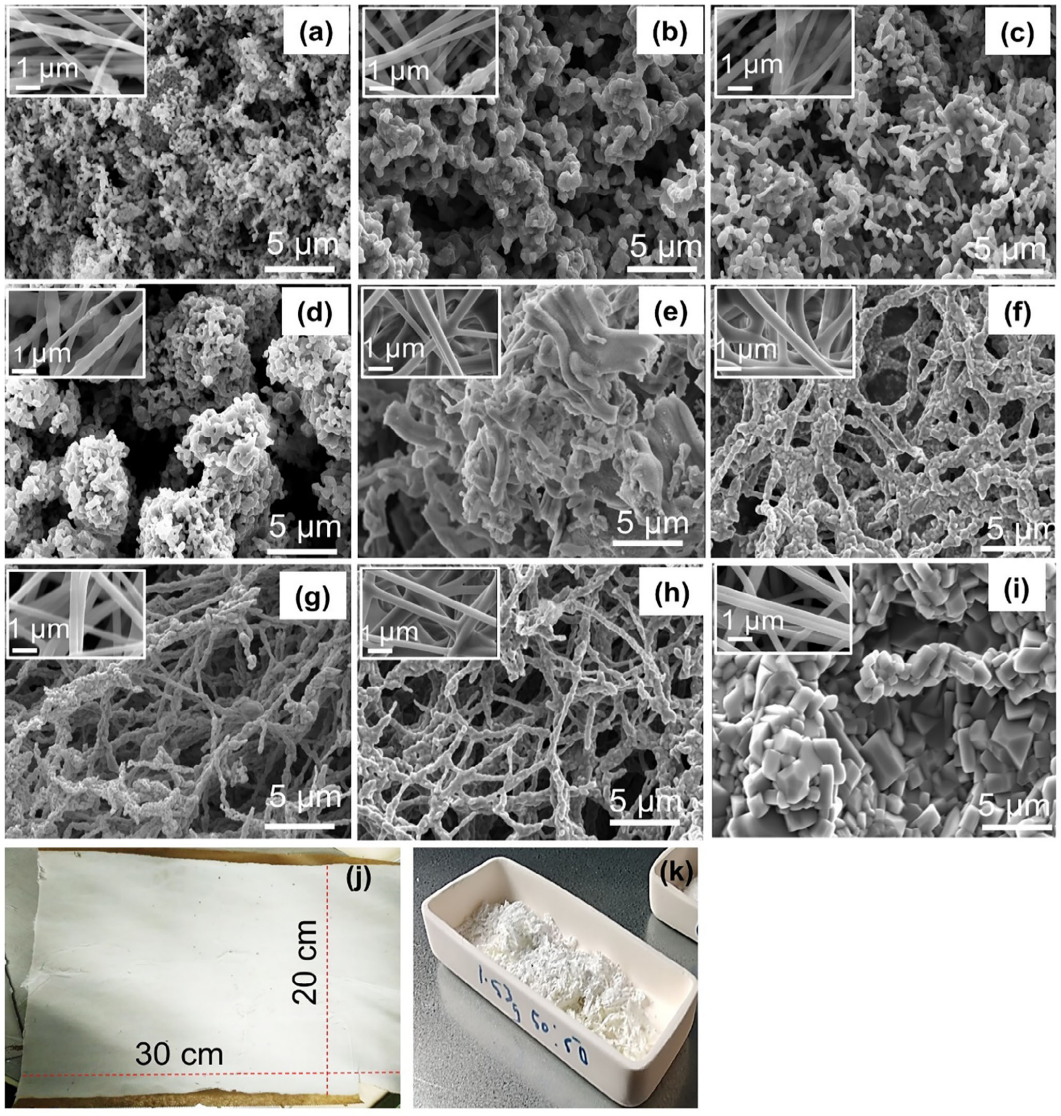
FE‐SEM micrographs of LATP‐NF a) ES2a, b) ES2b, c) ES3a, d) ES3b calcined at 800 °C and ES5 calcined at e) 600, f) 700, g) 750, h) 800, i) 900 °C, j) as prepared LATP mat and (k) corresponding LATP‐NF (after calcination) and all inset pictures represent the FE‐SEM micrographs of as spun LATP fibers.

LATP‐NFs were synthesized using various non‐toxic solvents (water, ethanol, and isopropanol) and polymers—poly(vinylpyrrolidone) (PVP) and PEO—via the electrospinning technique. The solvents, polymer types, and electrospinning parameters were systematically optimized. Based on the concentrations of the polymer, LATP precursors, and solvents, the electrospinning solutions (ES) were designated as ES1, ES2 (a and b), ES3 (a and b), ES4, and ES5. Detailed descriptions are provided in the Supporting Information (Experimental Section, Figures S1 and S2, and Table S1, Supporting Information). PVP, a commonly used polymer matrix for fabricating ceramic nanofibers, was initially tested for preparing LATP‐NF by dissolving it in LATP precursor solutions. Electrospinning solutions (ES1) prepared with water and ethanol as solvents failed to produce nanofibers, even after adjusting the PVP concentration (Figure S1a–d, Supporting Information). To address this, acetic acid was added to the water–ethanol mixture (ES2), which successfully yielded nanofibers (Figure S1e–i, Supporting Information). However, these fibers contained numerous beads and lacked a uniform fiber mat, despite variations in polymer and LATP solution concentrations. Hence, ES3 was formulated by substituting acetic acid with isopropanol. Water was initially used because many metal salts or precursors (e.g., nitrates) employed in LATP synthesis were highly soluble in water, making it a suitable solvent. However, PVP had limited solubility in water, particularly at higher concentrations. Additionally, water's high surface tension and low volatility hindered electrospinning, often resulting in bead formation instead of smooth fibers. Therefore, acetic acid was introduced as a co‐solvent to improve PVP solubility, lower the solution's surface tension, and enhance conductivity and viscosity control, thereby promoting uniform fiber formation. Nevertheless, its slower evaporation rate during electrospinning led to wet fibers or bead formation and raised safety concerns. Subsequently, isopropanol was introduced to further optimize the electrospinning conditions due to its lower dielectric constant and boiling point compared with water or acetic acid, improving the volatility of the spinning solution. This facilitated faster solvent evaporation, which promoted the formation of continuous fibers. This configuration, with varied LATP and PVP concentrations, produced more uniform nanofibers. However, microbeads were still observed in the resulting LATP mats (Figure S1j–m, Supporting Information), likely due to Ti‐precipitate formation in the electrospinning solution. All electrospun nanofiber mats were calcined at 800 °C for 4 h following a specific heating program (Figure S2l, Supporting Information), resulting in LATP‐NFs (Figure S1 n,o, Supporting Information). The microstructure of the LATP‐NFs was significantly influenced by the choice of polymer and solvent. Notably, PVP‐based nanofibers transformed into particles after calcination (**Figure** **1**a–d).

Consequently, PVP was replaced with PEO while retaining the solvents used in ES3 to fabricate ES4 and ES5. ES4 did not produce nanofibers due to the formation of a Ti‐precipitate‐like solution (Figure S2a–d, Supporting Information). After numerous trials and adjustments to the LATP precursor salts, a transparent and stable LATP precursor solution was successfully formulated for electrospinning (Figure S2e–g, Supporting Information). The ES5 solution, containing 4 wt.% PEO, successfully produced smooth, bead‐free nanofibers, as shown in Figure 1e–i (inset) and j, and Figure S2h–j (Supporting Information). The calcination temperature was optimized using a specific heating program ranging from 600 °C to 900 °C (Figure S2l, Supporting Information).

**Figure 2:**
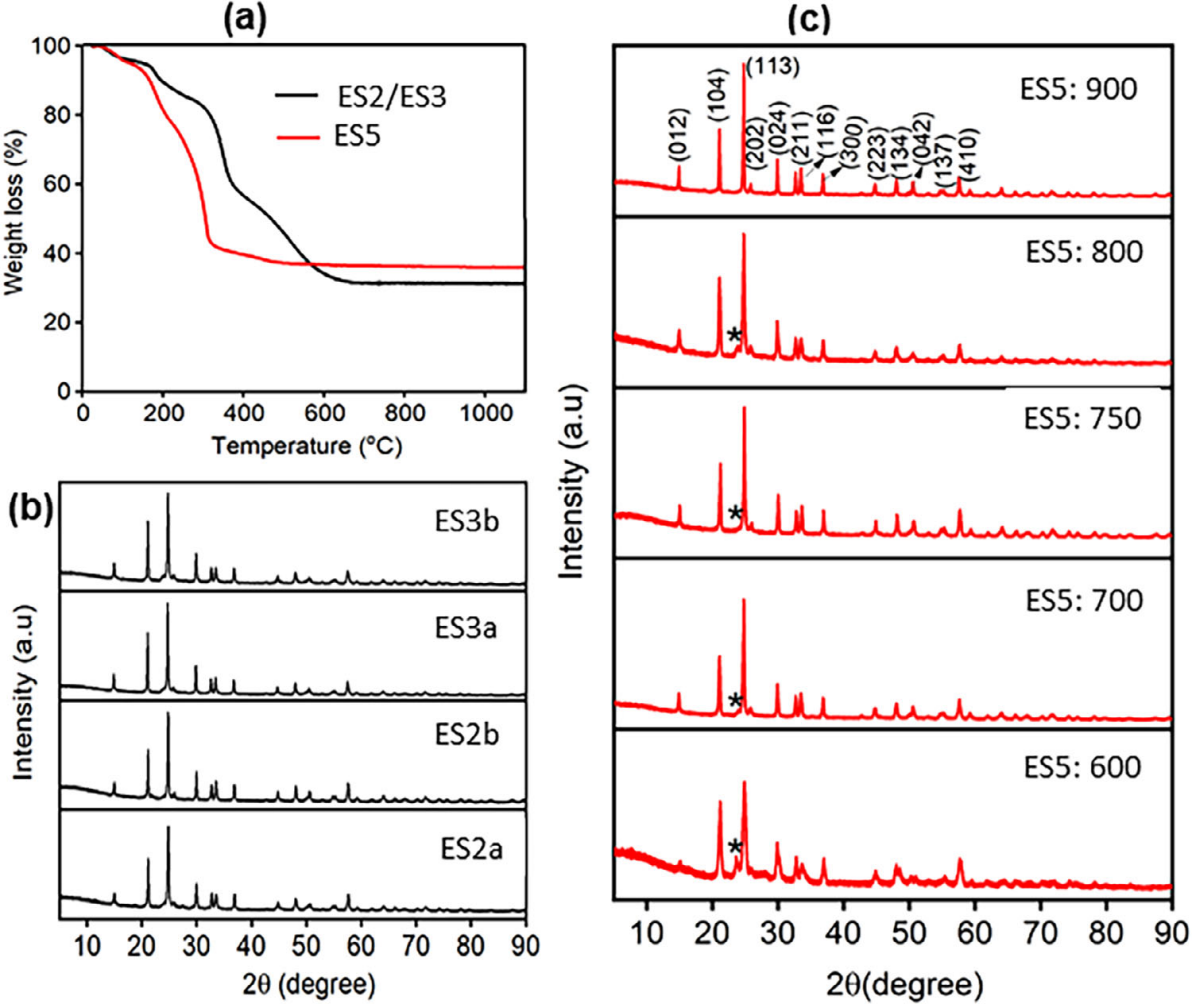
a) TGA of as‐prepared LATP mat; b) XRD pattern of LATP‐NF prepared by ES2 and ES3 with PVP polymer, and c) XRD pattern of LATP‐NF prepared by ES5 using PEO with different calcination temperatures (^*^ impurity phase of Li_4_P_2_O_7_).

Nanofibers calcined at 600 °C exhibited agglomeration and incomplete structures (Figure 1e), whereas calcination at 700–800 °C produced continuous ceramic nanofiber networks with minimal grains and grain boundaries (Figure 1f–h). In contrast, calcination at 900 °C resulted in the formation of microparticles instead of nanofibers (Figure 1i). The optimal calcination range of 700–800 °C yielded robust and continuous fibrous ceramic networks with an average diameter of 150–250 nm. The heating program and calcination temperature were critical for achieving smooth ceramic nanofibers. A slow heating rate was beneficial, likely minimizing fiber shrinkage, collapse, and cracking, thereby ensuring uniform fiber formation. The PEO‐based LATP electrospinning solution produced better ceramic nanofibrous microstructures than other formulations. The polymer type and concentration of LATP precursors also influenced the final product of LATP‐NF, as shown in the TGA curves (**Figure** **2**a; Figure S2k, Supporting Information). The weight loss observed between 25 and 200 °C is attributed to the initiation of metal‐organic complex reactions (hydrolysis and condensation), which facilitate precursor gelation within the fibers.

A significant weight loss occurred between 250–400 °C and 400–600 °C, corresponding to the decomposition of the polymer matrices (PEO and PVP) into gaseous products such as H_2_O, CO_2_, and CO. Beyond 600 °C, no substantial weight loss was observed, indicating that the LATP precursor compounds (e.g., lithium, aluminum, titanium, and phosphate species) reacted to form an intermediate amorphous LATP phase that subsequently crystallized upon calcination. The yield of LATP‐NF obtained from the ES5 method was considerably higher than that from ES2 and ES3.

The XRD pattern of LATP‐NF prepared using PVP polymer with different electrospinning solutions (ES2a, ES2b, ES3a, and ES3b) is shown in Figure 2b. The diffraction peaks were indexed to the crystallographic planes of NASICON‐type LATP, confirming the rhombohedral crystal structure of LATP‐NF with the *R‐3c* space group.^[^ ^24^, ^25^ ^]^ Figure 2c shows the XRD patterns of LATP‐NF synthesized using ES5 at different calcination temperatures. The ceramic nanofibers calcined at 750 °C and above exhibited diffraction peaks corresponding to a rhombohedral crystal structure, along with a minor impurity peak (*). However, samples calcined at 600 °C displayed broader reflections and a noticeable impurity peak at 23.45°, attributed to the Li_4_P_2_O_7_ phase (*), indicating incomplete reactions among the LATP precursor salts. Calcination at 750–800 °C was determined to be optimal for achieving nanocrystalline LATP‐NFs.

### 2.2 Morphological, Optical, and Mechanical Properties of LATP‐NF Composite Solid Electrolytes

Based on the morphological, structural, and TGA results, the ES3a, ES3b, ES5:600, ES5:700, ES5:750, and ES5:800 samples were selected as ceramic nanofibers (Figure S3 and Table S2, Supporting Information) for fabricating CPE films. The composition of PVDF and LiTFSI was maintained at 60:40 wt.% for the solid polymer electrolyte (SPE), while the ratio of PVDF–LiTFSI to LATP‐NF was fixed at 85:15 wt.% to investigate the effect of the LATP‐NF microstructure on the electrochemical performance of the CPEs. The results clearly highlighted the influence of LATP particles and the nanofibrous structure on the physicochemical properties of the CPEs. Photographs and FE‐SEM micrographs of the SPE and CPEs are shown in Figures S4 and S5 (Supporting Information). The SPE exhibited several micropores, which decreased upon the incorporation of LATP‐NFs (Figure S5, Supporting Information). The CPE‐ES3a and CPE‐ES3b samples led to particle aggregation and the formation of small pores (Figure S5b,c, Supporting Information). The CPE‐ES5:600 ceramic nanofibers also showed uneven dispersion within the CPE due to their aggregated and thick ceramic fibers (Figure S5d, Supporting Information). In contrast, CPE‐ES5:750 demonstrated a relatively uniform distribution of ceramic nanofibers with fewer visible pores than the other samples (Figure S5f, Supporting Information).

The TGA curves of the CPEs, shown in Figure S6a (Supporting Information), revealed no significant weight loss between 25 and 200 °C, confirming the negligible presence of residual solvent and its minimal effect on thermal stability. A major weight loss between 350 and 450 °C corresponded to the melting and decomposition of the PVDF polymer and the LiTFSI salt.^[^ ^26^, ^27^ ^]^ The incorporation of LATP‐NFs into the SPE enhanced the thermal stability of the CPE films. The bulk resistance was determined from the Nyquist plots by identifying the high‐frequency intercept on the real axis, and the calculated ionic conductivities are summarized in Table S3 (Supporting Information). Among the samples, CPE‐ES5:750 exhibited the highest ionic conductivity of 1.6 × 10^−^⁴ S cm^−1^, surpassing the other CPEs. Based on the microstructural features and ionic conductivities of the CPEs, ES5:750 ceramic nanofibers were selected for further optimization to evaluate the effects of their concentration on the electrochemical performance of the CPEs.

**Figure 3:**
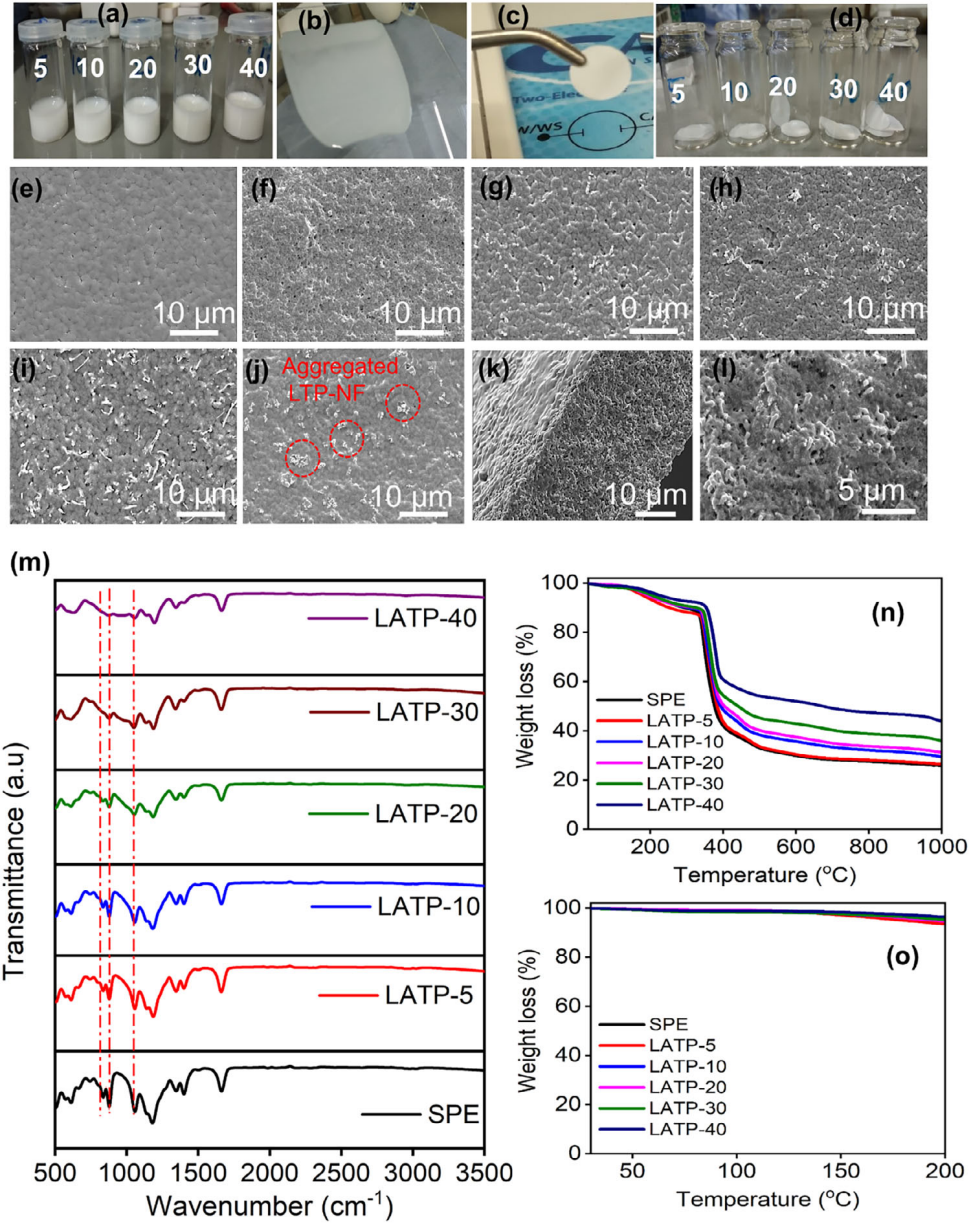
Digital Photographs: a) CPE slurries, b) as coated CPE film, c and d) dried CPE films; FE‐SEM micrographs of CPE films: e) SPE, f) LATP‐5, g) LATP‐10, h) LATP‐20, i) LATP‐30, j) LATP‐40 and k,l) cross‐sectional FE‐SEM micrographs of LATP‐30 film; m) FTIR spectra, n)TGA and o) enlarged TGA (25‐200 °C) curves of SPE and CPE films with different concentration of ES5:750.

The compositions of ES5:750, PVDF, and LiTFSI are listed in Table S4 (Supporting Information). Hereafter, CPEs with different ES5:750 concentrations (wt.%) were denoted as LATP–X, where X represents the wt.% of ES5:750 (5, 10, 20, 30, and 40 wt.%). Digital images of the CPE slurries and coated films are presented in **Figure** **3**a,b, confirming the formation of bubble‐free, homogeneous coatings on glass plates using an electrode coating machine. Highly flexible CPE films were obtained after solvent evaporation (Figure 3c,d). The microstructures of the SPE and CPEs with varying ES5:750 concentrations are shown in Figure 3e–j. The SPE displayed a porous morphology, but pore density decreased with the addition of LATP ceramic nanofibers. At lower concentrations (up to 10 wt.%), the distribution of LATP nanofibers was less prominent (Figure 3f,g), whereas LATP‐30 exhibited a more uniform distribution of ceramic nanofibers (Figure 3i). Cross‐sectional FE‐SEM images of LATP‐30 demonstrated that ES5:750 achieved a consistent morphology and uniform dispersion within the polymer matrix (Figure 3k,l). The combination of bath ultrasonication for gentle pre‐dispersion and brief probe sonication for intensive mixing prevented fiber damage while achieving a stable, uniform LATP‐NF dispersion at a maximum loading of 30 wt.%. However, higher concentrations (LATP‐40) resulted in nanofiber aggregation (Figure 3j).

Fourier transform infrared (FTIR) spectroscopy was employed to analyze the interactions between the LATP ceramic nanofibers and the polymer matrix (Figure 3m). The vibrational peaks at 616, 672, 742, 834, 878, 1060, 1184, 1334, 1397, and 1659 cm^−1^ were attributed to the characteristic modes of PVDF–LiTFSI.^[^ ^26^, ^27^, ^28^, ^29^ ^]^ The vibrational bands at 1020–1040 cm^−1^ corresponded to the crystalline phase of PVDF,^[^ ^29^ ^]^ while the peaks at 1334 and 1397 cm^−1^ were assigned to the stretching vibrations of the CH_2_ groups. The peaks between 1150 and 1240 cm^−1^ arose from the stretching vibrations of C─F bonds.^[^ ^29^ ^]^ The band observed at 1659 cm^−1^ was attributed to TFSI^−^ ions trapped within the PVDF polymer matrix.^[^ ^26^ ^]^ The vibrational peaks between 500 and 700 cm^−1^ were associated with the TiO_6_ stretching modes, whereas those between 900 and 1300 cm^−1^ corresponded to P─O bonds. Peaks below 500 cm^−1^ were assigned to PO_4_ stretching and bending vibrations of LATP.^[^ ^30^, ^31^ ^]^ The intensities of the characteristic polymer peaks at 834 and 878 cm^−1^, as well as the crystalline peak at 1020 cm^−1^, decreased as the LATP‐NF concentration increased, indicating enhanced amorphous character in the CPEs. The interaction between the LATP‐NFs and the polymer matrix likely facilitated the immobilization of TFSI^−^ anions via Lewis acid–base interactions. This, in turn, promoted the dissociation of LiTFSI salt and improved Li⁺ migration within the PVDF and LATP‐NF/PVDF interface. The thermal stability of the SPE and the CPEs with different ES5:750 concentrations was further examined by TGA analysis (Figure 3n,o). The CPE films exhibited negligible weight loss up to 150 °C and a minor weight loss of ≈4% up to 200 °C (Figure 3o), confirming their thermal stability under normal battery operating conditions. A significant weight loss was observed between 300 and 450 °C due to the decomposition of the polymer and salt components.^[^ ^5^ ^]^ However, the residual mass of the CPEs increased with increasing LATP‐NF concentration (Figure 3n), indicating improved thermal stability of the CPE films.

**Figure 4:**
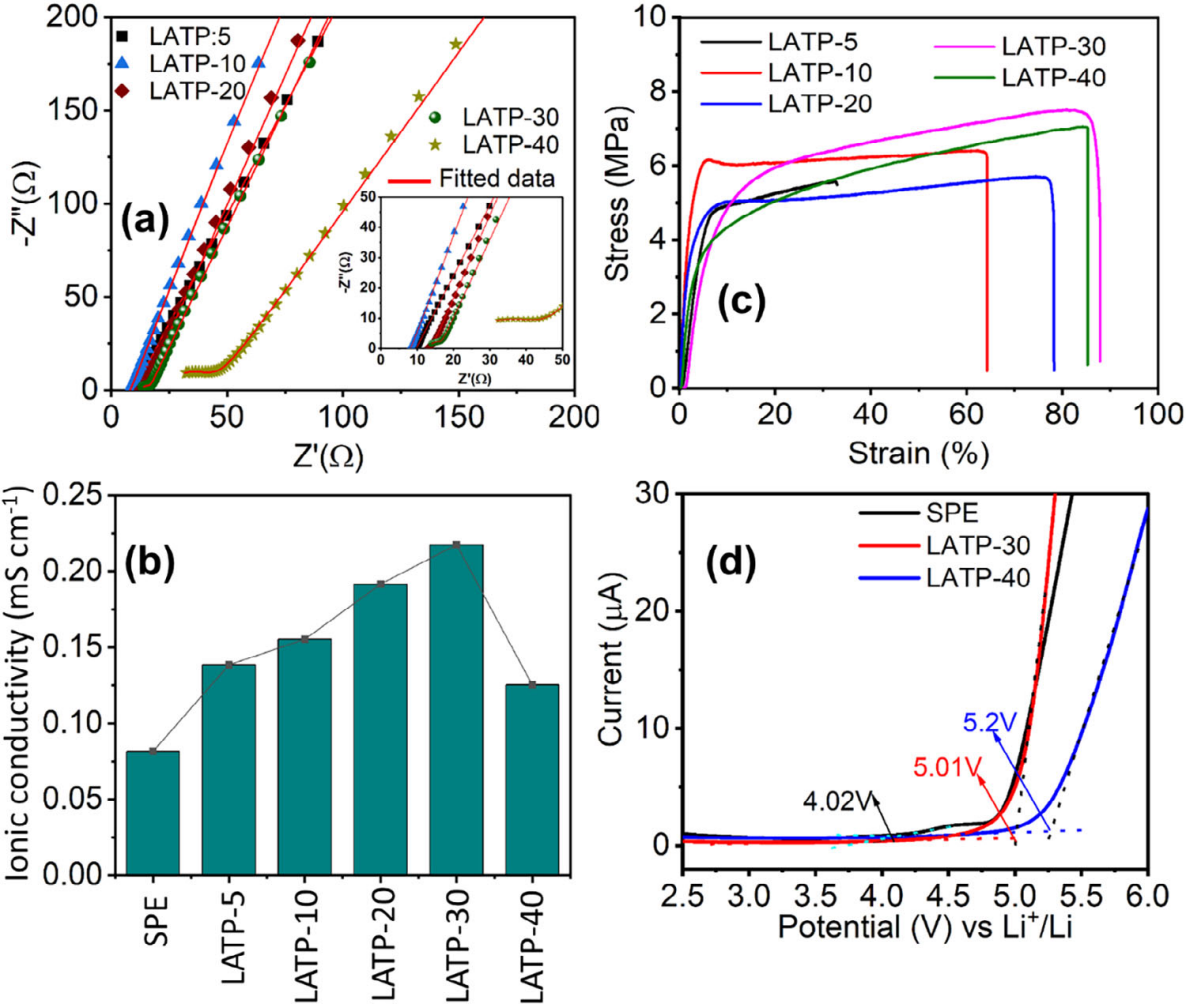
a) Nyquist plots, b) comparison of ionic conductivity, c) strain versus stress curves, and d) LSV curves of SPE and CPE films with different concentrations of ES5:750 ceramic nanofibers.

The intrinsic properties of the CPEs and SPE, such as the electrochemical stability window (measured by LSV) and ionic conductivity (measured by EIS), were evaluated without the presence of a liquid electrolyte. The bulk resistances of the SPE and CPEs were determined from Nyquist plots (**Figure** **4**a; Figure S6c, Supporting Information). The first intercept of the semicircle with the real axis (Z′) in the high‐frequency region represented the bulk resistance of the solid electrolytes.^[^ ^32^ ^]^ The low‐frequency region was typically associated with other processes such as ion blocking or electrode polarization.^[^ ^32^ ^]^ All EIS data were fitted using equivalent circuit models (Figure S8 and Table S5, Supporting Information) in EC‐Lab software to determine the accurate bulk resistance. The fitted data were consistent with the experimental values. In the equivalent circuit, R_b_ represented the bulk resistance, R_int_ denoted interfacial or grain boundary resistance, CPE described the constant phase element corresponding to non‐ideal capacitance, and *n* was the constant. The ionic conductivity of each sample was calculated based on its respective bulk resistance and film thickness (Table S6, Supporting Information). LATP‐5 and LATP‐10 showed no semicircles in the Nyquist plots, indicating that ionic migration was primarily governed by the polymer matrix due to the lower LATP content (5 and 10 wt.%). The low concentrations of LATP‐NFs may not have integrated effectively with the polymer matrix, hindering ionic transport. However, LATP‐5 and LATP‐10 exhibited lower bulk resistances and higher ionic conductivities than the SPE. When the LATP‐NF concentration increased beyond 10 wt.%, the Nyquist plots displayed a slightly depressed semicircular arc in the high‐frequency region, indicative of grain boundary or interfacial impedance between the LATP‐NFs and the polymer matrix.

The LATP‐30 sample exhibited a higher ionic conductivity of 0.218 mS cm^−1^ compared with all other LATP compositions and was one order of magnitude higher than that of the SPE (0.0816 mS cm^−1^) at RT. An optimal amount of LATP‐NFs likely improved the LATP–polymer interface and increased the amorphous regions of the polymer matrix, thereby enhancing the ionic conductivity of the CPE film (Figure 4b). However, higher LATP concentrations, as in LATP‐40, resulted in a noticeable semicircle in the high‐frequency region, attributed to aggregated LATP‐NFs or increased interfacial resistance at the LATP–polymer interface, which reduced ionic conductivity to 0.125 mS cm^−1^. An excessive amount of LATP nanofibers resulted in agglomeration and diminished flexibility, which consequently hindered ion transport within the polymer matrix. This trend indicated that LATP‐30 represented the optimal ratio to the polymer matrix for achieving low resistance and high conductivity. EIS measurement was conducted at various temperatures to determine the activation energy (*E_a_*) of the SPE and the optimized LATP‐30 (Figure S7 and Table S7, Supporting Information). LATP‐30 exhibited a lower activation energy (0.27 eV) than the SPE (0.39 eV), suggesting that ion transport occurred more easily through the nanofibrous LATP structure. The enhanced ionic conductivity was attributed to the high surface area and interconnected ion transport pathways of the LATP nanofibers, which facilitated faster lithium‐ion migration relative to the polymer matrix in the SPE.

The mechanical stability of the CPE films was evaluated using five samples for each concentration (Figure S9, Supporting Information; Figure 4c). Variations in the stress–strain curves with increasing LATP‐NF content were attributed to the mechanical reinforcement provided by the ceramic nanofibers and their interaction with the polymer matrix. The LATP‐NFs, being inherently rigid and mechanically robust, acted as load‐bearing reinforcements within the polymer matrix, transferring stress from the softer PVDF matrix to the stiffer LATP fibers. This resulted in higher mechanical strength, enabling the composite to withstand greater applied forces. At low filler concentrations, the LATP‐NFs were insufficient to effectively transfer stress across the polymer–ceramic interface. As the LATP content increased, more nanofibers participated in stress transfer, thereby enhancing the composite's load‐bearing capacity. Consequently, the optimal LATP‐30 composition improved the toughness of the CPE by enhancing the matrix's ability to deform before failure. The LATP nanofibers might have interacted with the PVDF matrix and LiTFSI salt, which could have improved polymer chain alignment and overall mechanical properties, thereby contributing to the enhanced stress and strain performance. The LSV curves of Li|SPE|SS and Li|LATP‐30&40|SS cells are presented in Figure 4d. LATP‐30 and LATP‐40 exhibited wider electrochemical stability windows (ESWs) of 5.01 and 5.2 V, respectively, compared with the SPE (4.02 V). LATP, an inorganic solid electrolyte with an inherently wide ESW (≈5 V),^[^ ^33^ ^]^ contributed its intrinsic stability to the CPE when incorporated into the polymer matrix, particularly at higher LATP contents (30–40 wt.%), thereby increasing the overall ESW. This enhancement rendered the CPEs more resistant to high‐voltage decomposition.

### 2.3 Influence of LATP Nanofibrous Microstructure on Lithium Stripping/Plating and Electrochemical Performance

**Figure 5:**
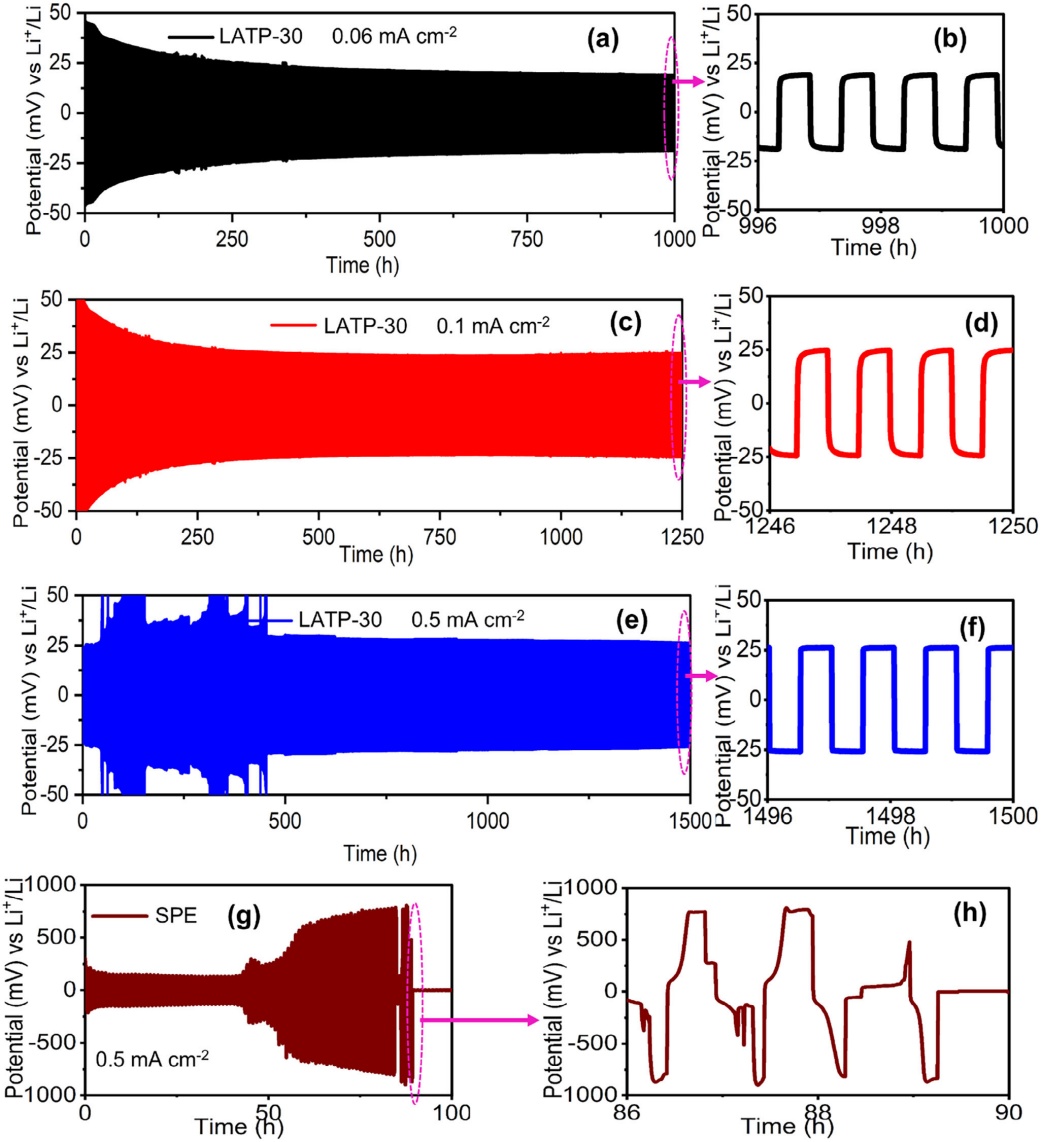
Long‐term lithium platting/stripping cycling (GCD) curves of Li|LATP‐30|Li cells: a) at 0.06 mA cm^−2^ for 1000 cycles, c) at 0.1 mA cm^−2^ for 1250 cycles and e) at 0.5 mA cm^−2^ for 1500 cycles and b, d, f) corresponding enlarged GCD curves; g) Lithium platting/stripping cycling of Li|SPE|Li cell at 0.5 mA cm^−2^ and h) corresponding enlarged curves.

Based on the microstructural, ionic conductivity, thermal, and mechanical properties, LATP‐30 was identified as the optimal sample for further investigation of lithium plating/stripping, electrochemical stability, rate capability, and cycling performance. Long‐term lithium stripping and plating tests of Li|LATP‐30|Li symmetric cells were conducted at various current densities (0.06, 0.1, and 0.5 mA cm^−2^), as shown in **Figure** **5**a–f. The LATP‐30 cells exhibited excellent lithium plating and stripping performance at 0.06 and 0.1 mA cm^−2^ over 1000–1250 cycles, with overpotentials of 19 and 24 mV, respectively. In addition, the LATP‐30 cell operated stably at a high current density of 0.5 mA cm^−2^ for more than 1500 cycles without failure (Figure 5e,f). Minor noise in some curves was attributed to a transient soft short circuit, which stabilized after a few cycles. The LATP‐30 symmetric cell initially showed an overpotential of ≈100–125 mV up to 125 cycles and 40–50 mV up to 300 cycles, which gradually decreased and stabilized at ≈27 mV after 1500 cycles (Figure S10, Supporting Information). During the early cycles, the relatively higher overpotential likely originated from incomplete wetting and the formation of an initial solid–electrolyte interphase (SEI) between the lithium metal and LATP‐30 CPE. As cycling proceeded, the small quantity of liquid electrolyte retained at the interfaces facilitated improved wetting and infiltration within the polymer–ceramic matrix, thereby enhancing ionic contact and minimizing interfacial resistance. In contrast, the SPE cell exhibited a gradual arc in the voltage plateau after the 45th cycle, a sharp increase after the 50th cycle, and short‐circuited after 88 cycles at 0.5 mA cm^−2^ (Figure 5g,h). This failure was attributed to non‐uniform lithium plating/stripping, which led to uneven surface deposition and eventual dendrite formation and penetration. These results demonstrated that the well‐interconnected LATP‐NFs (ES5:750) with liquid electrolyte at the interface (Li|CPE) acted as a reinforcing skeleton, effectively preventing dendrite penetration and ensuring electrochemical stability during extended cycling.

**Figure 6:**
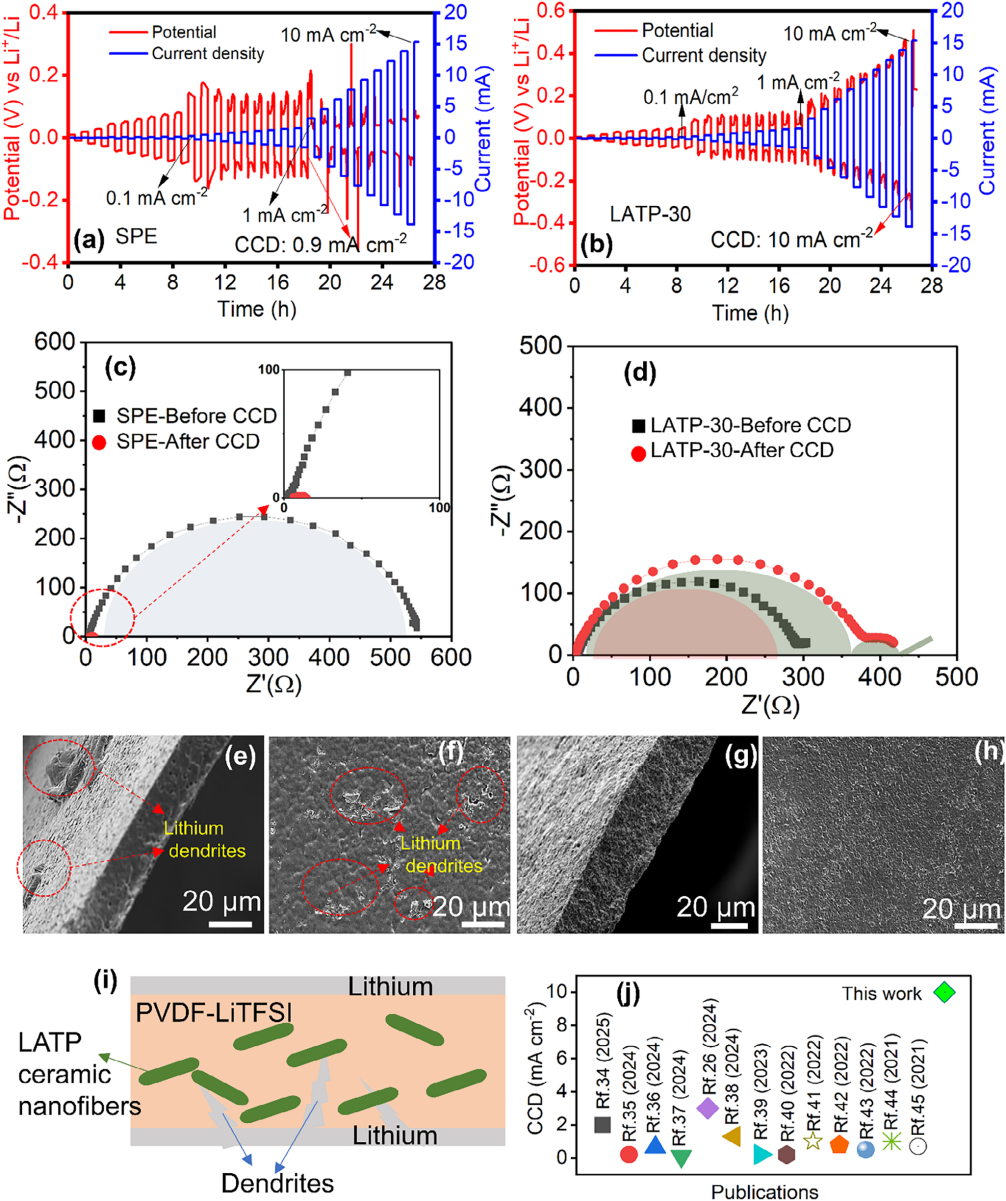
Rate performance/CCD test of the cells: a)Li|SPE|Li and b) Li|LATP‐30|Li symmetric cells; c, d) EIS of before and after CCD test; cross‐sectional and surface FE‐SEM micrographs of e and f) SPE and g and h) LATP‐30 after CCD test, i) schematic illustration of the dendrite suppression mechanism in the CPE and j) comparison of CCD values or maximum current density reported in the literature for LATP based CPEs.

The critical current density (CCD)—defined as the maximum current density at which stable lithium electrodeposition occurred without dendritic short‐circuiting—was evaluated for Li|SPE|Li and Li|LATP‐30|Li symmetric cells. The rate performance was measured by incrementally increasing the current density from 0.01 to 10 mA cm^−2^ in steps of 0.01, 0.1, and 1 mA cm^−2^, as shown in **Figure** **6**a,b. Rapid lithium dendrite growth through the SPE led to short‐circuiting at a low CCD of 0.9 ± 0.1 mA cm^−2^ (Figure 6a). EIS analysis before and after the CCD test revealed a distinct high‐frequency semicircle for the SPE before testing, with a charge‐transfer resistance (*R_ct_*) of 540 Ω. After the CCD test, the resistance dropped abruptly to ≈0 Ω, indicating a short circuit (Figure 6c).

In comparison, the Li|LATP‐30|Li cell exhibited an excellent CCD of 9.5 ± 0.5 mA cm^−2^ with a capacity of 5 mAh cm^−2^ (Figure 6b), confirming the superior rate performance of the LATP‐30 CPE. Figure 6d illustrates the EIS spectra of the cell before and after the CCD test. Prior to the CCD test, the Li|LATP‐30|Li cell exhibited a high‐frequency semicircle with a charge‐transfer resistance (*R_ct_*) of 280 Ω. Following the CCD test, the cell displayed a high‐frequency semicircle along with an additional small semicircle in the low‐frequency region, indicating that the cell did not short‐circuit even under an applied current density of 10 mA cm^−2^. The *R_ct_* increased to 350 Ω after the CCD test, and the small semicircle observed at low frequency corresponded to the interfacial resistance (80 Ω) arising from interactions between the polymer/LATP‐NF and the mixed conductive interphase (Figure 6d). Cross‐sectional and surface FE‐SEM micrographs of the SPE after the CCD test (Figure 6e,f; Figure S11, Supporting Information) revealed lithium dendrite penetration and the presence of dead lithium, which correlated with the sudden drop in resistance observed in the EIS spectra after the CCD test. LATP‐30 exhibited no evidence of dendrite penetration or dead lithium on either the surface or cross‐section of the film, as shown in Figure 6g,h. The incorporation of nanofibrous LATP‐NF fillers into the CPE played a pivotal role in preventing dendrite penetration and enhancing the CCD (Figure 6i). Lithium dendrites typically grow as needle‐like structures that attempt to pierce through the electrolyte. The LATP‐NFs acted as mechanical barriers, redistributing and absorbing the stress induced by dendrite growth, as supported by the EIS and FE‐SEM results obtained after the CCD test. Additionally, the combination of LATP‐NFs and a small amount of liquid electrolyte (LE) improved interfacial contact and ion diffusion within the CPE, reducing polarization and enabling stable operation at a high current density of 10 mA cm^−2^. The achieved CCD value for the LATP‐30 CPE surpasses those of recently reported LATP particle‐ and nanofiber‐based CPEs (Figure 6j).^[^ ^26^, ^34^, ^35^, ^36^, ^37^, ^38^, ^39^, ^40^, ^41^, ^42^, ^43^, ^44^, ^45^ ^]^ A comprehensive comparison of the CCD results from this work with literature data is summarized in Table S8 (Supporting Information).

**Figure 7:**
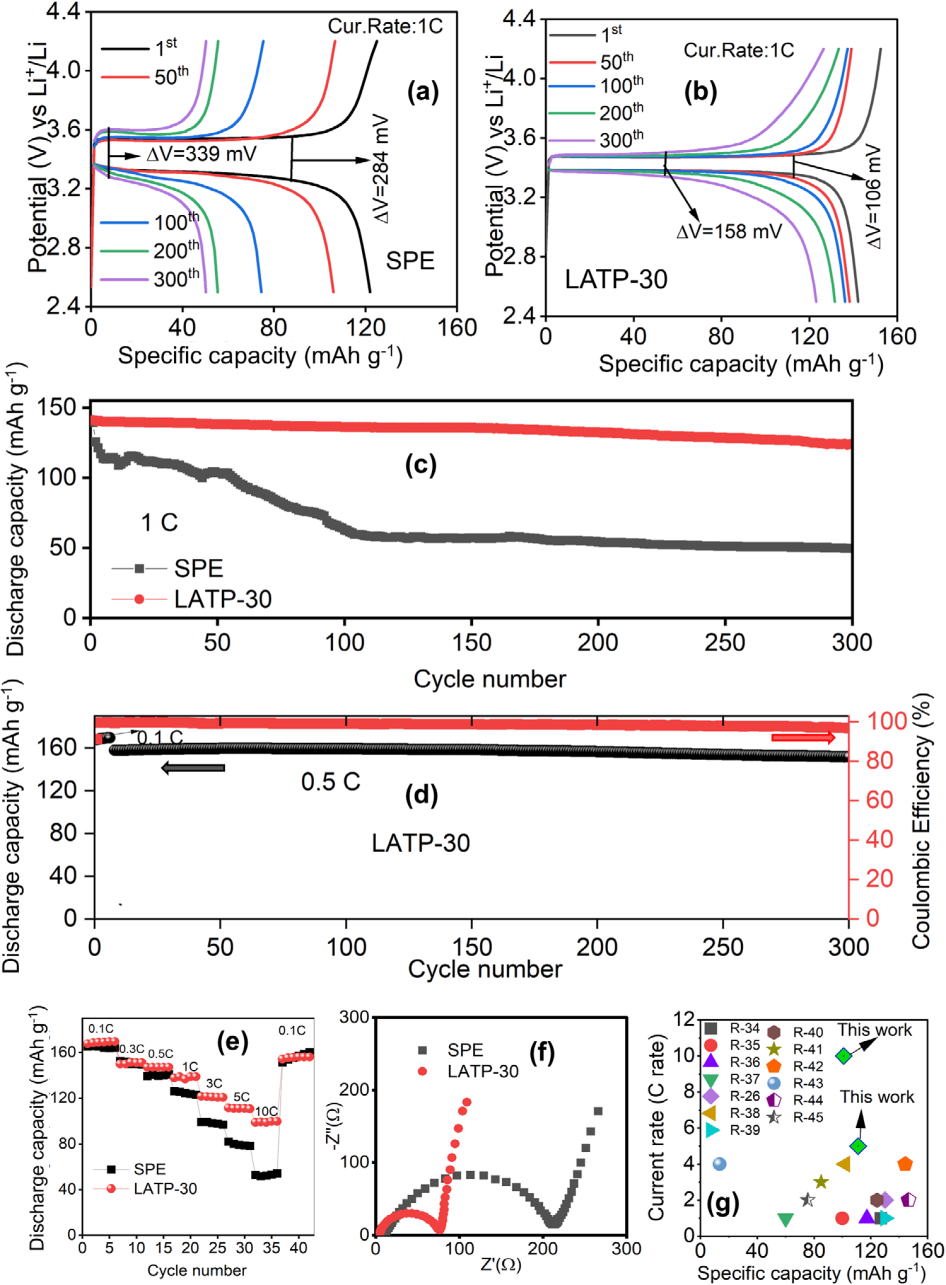
Potential versus specific capacity curves of a) LFP|SPE|Li and b) LFP|LATP‐30|Li cells, c) long‐term cycling stability of SPE and LATP‐30 cells at 1 C rate, d) long‐term cycling stability and coulombic efficiency of LATP‐30 cell at 0.5 C rate, e) rate performance, f) EIS spectra and g) comparison of the discharge capacity at maximum rate/rate performance of this work with recent literature reports.

The electrochemical performance of LFP|SPE|Li and LFP|LATP‐30|Li coin cells was evaluated within a potential range of 2.5–4.2 V at 25 °C (**Figure** **7**a,b). The charge–discharge voltage plateau for the SPE decreased significantly with increasing cycle number, as shown in Figure 7a. The potential differences between the charge and discharge plateaus for the 1st and 300th cycles were determined to be 284 and 339 mV, respectively, indicating high polarization in the SPE cells. This decline in voltage plateau across successive cycles was attributed to the interfacial instability of the SPE in contact with lithium metal.

Incorporation of LATP‐NFs into the CPE played a crucial role in maintaining a stable charge–discharge plateau and minimizing the potential difference between charge and discharge curves, even after 300 cycles (1st cycle: 106 mV; 300th cycle: 158 mV) (Figure 7b). The incorporation of liquid electrolyte at the Li|CPE and LFP|CPE interfaces facilitates efficient lithium‐ion transport and suppresses the formation of inactive lithium, thereby enabling more reversible lithium cycling. It also mitigated concentration polarization through faster ionic transport within the liquid electrolyte–polymer matrix/LATP‐NF composite and reduced charge‐transfer polarization by improving electrode–electrolyte interactions. This reduction in polarization contributed to a stable and flat voltage plateau during prolonged cycling. The long‐term cycling stability of the LFP|SPE|Li and LFP|LATP‐30|Li cells is shown in Figure 7c. For the SPE cell, the initial charge–discharge capacities were 128 and 120 mAh g^−1^, which decreased to 50 and 48 mAh g^−1^ after 300 cycles at a current rate of 1C. The SPE exhibited rapid capacity fading after 70 charge–discharge cycles. In contrast, the LATP‐30 cell displayed initial charge–discharge capacities of 153 and 143 mAh g^−1^, and retained 127 and 123 mAh g^−1^ after 300 cycles—corresponding to 86 % capacity retention (Figure 7c) at 1C. Figure 7d shows the long‐term cycling performance of the LATP‐30 cell at 0.5C. During cell formation (five cycles), it delivered a high discharge capacity of 169 mAh g^−1^ at 0.1C with a coulombic efficiency of 99.65%, operating close to the theoretical specific capacity of LFP (170 mAh g^−1^). At a moderate current density (0.5C), the cell achieved an initial specific discharge capacity of 159 mAh g^−1^ and retained 153 mAh g^−1^ after 300 cycles, corresponding to 96.22% capacity retention. The incorporation of LATP‐NF into the CPE significantly enhanced capacity retention in the LATP‐30 cells. The ceramic fibrous structure formed a 3D continuous network that enabled uniform lithium‐ion transport across the electrolyte, reducing ionic concentration polarization, especially under high current densities (1C). Moreover, LATP nanofibers enhanced the ionic conductivity of the composite LFP cathode, improved electrochemical reactions, and mitigated capacity fading during extended cycling. At a lower rate (0.5C), the slower cycling provided lithium ions more time to diffuse, further minimizing polarization. Thus, the compatibility of LATP with LFP cathode chemistry effectively reduced capacity fade and supported high retention during long‐term operation.

The rate performance of LFP|SPE|Li and LFP|LATP‐30|Li cells was evaluated at various charge/discharge rates ranging from 0.1C to 10C (Figure 7e). Five charge–discharge cycles were performed at each C‐rate to ensure stability and consistency. The SPE cell exhibited poor rate capability beyond 0.3C, and it showed a sharp capacity drop at rates above 3C due to high interfacial resistance and slow ion transport in the polymer‐only matrix. The LATP‐30 cell demonstrated superior rate performance, especially at higher C‐rates (>0.5C), delivering discharge capacities of 122, 111, and 101 mAh g^−1^ at 3C, 5C, and 10C, respectively. Across moderate to high C‐rates (1C–10C), the LATP‐30 cell consistently outperformed the SPE cell in specific capacity. Notably, when the rate was returned to 0.1C, the LATP‐30 cell nearly recovered its initial capacity, indicating robust structural and electrochemical stability after high‐rate cycling.

These results were benchmarked against recent reports on LATP particle‐ and nanofiber‐based CPEs that incorporate liquid electrolyte at the interfaces (Figure 7g; Table S9, Supporting Information).^[^ ^26^, ^34^, ^35^, ^36^, ^37^, ^38^, ^39^, ^40^, ^41^, ^42^, ^43^, ^44^, ^45^ ^]^ As summarized in Table S9 (Supporting Information) and Figure 7g, only a few studies^[^ ^26^, ^42^, ^43^ ^]^ reported rate performance at 4C, typically with low specific capacities. The present study surpassed these reports, achieving the highest recorded C‐rate of 10C with a specific capacity of 101 mAh g^−1^ for the LATP‐30 CPE. The enhanced CCD and rate performance of the CPE containing 30 wt.% LATP‐NF could be attributed to both improved ionic conductivity and enhanced Li⁺ transport pathways, and mechanical robustness arising from the fibrous LATP‐NF architecture. The continuous 1D LATP nanofibers formed an interconnected ion‐conducting network within the PVDF–LiTFSI matrix, providing direct and continuous Li⁺ migration channels, reducing interfacial resistance, and facilitating uniform Li⁺ flux at the interface. The combination of LATP‐NF and a small amount of liquid electrolyte (6 µL cm^−2^) improved interfacial contact and ion diffusion within the CPE, reducing polarization effects and enabling excellent rate capability. These findings established the LATP‐30 CPE as a promising electrolyte for high‐power lithium metal batteries requiring excellent C‐rate performance and long‐term stability. The Nyquist plots of LFP|SPE|Li and LFP|LATP‐30|Li cells after formation (five cycles at 0.1C) are shown in Figure 7f. Both cells displayed a high‐frequency semicircle and a low‐frequency spike, representing the charge‐transfer resistance (*R_ct_*) and Warburg impedance, respectively.^[^ ^5^ ^]^ The LATP‐30 cell exhibited a smaller semicircle, indicating lower charge‐transfer resistance (77 Ω) compared to the SPE cell (224 Ω). This improvement resulted from the synergistic combination of LATP nanofibers, flexible polymer matrix, and LE additive.

The electrochemical performance of LATP particles (30 wt.%) was further compared with LATP‐NF (Figure S12, Supporting Information). The LATP particle‐based cell exhibited significant capacity degradation and failed after 174 cycles. Consequently, the LATP‐30 (LATP‐NF) cell demonstrated superior cycling stability compared to both the LATP particle‐based CPE and the SPE cells. Additionally, the electrochemical performance of the LATP‐30 CPE was evaluated using a high‐voltage NMC811 cathode (Figure S13, Supporting Information). The NMC811|LATP‐30|Li cell exhibited initial charge and discharge capacities of 232 and 191 mAh g^−1^ at 0.1C, respectively, and maintained ≈82% capacity retention after 100 cycles at 1C. These results demonstrated that the CPE films possessed good stability under high‐voltage conditions. Overall, the LATP‐30 electrolyte exhibited high ionic conductivity, low interfacial resistance, and excellent long‐term cycling stability, making it a promising candidate for QSLMBs.

## 3 Conclusion

LATP ceramic nanofibers were successfully fabricated via electrospinning using a water‐based solvent system with two different polymers (PVP and PEO). The microstructure of the ceramic nanofibers was systematically optimized, achieving diameters ranging from 150 to 250 nm. The influence of the LATP‐NF nanofibrous microstructure and its concentration on the physicochemical and electrochemical properties of the CPE was systematically investigated. The LATP‐30 sample exhibited higher ionic conductivity (0.218 mS cm^−1^), a wider electrochemical stability window (5 V), and a greater critical current density (CCD) of 10 mA cm^−2^ compared with the SPE (0.0816 mS cm^−1^, 4 V, and 0.9 mA cm^−2^). The LATP nanofibrous structure, combined with the presence of liquid electrolyte at the Li|CPE interface, effectively suppressed lithium dendrite penetration into the electrolyte. Consequently, the LFP|LATP‐30|Li cell exhibited superior electrochemical performance, delivering a discharge capacity of 153 mAh g^−1^ after 300 charge–discharge cycles with an impressive capacity retention of ≈97% at 25 °C. Furthermore, the cell demonstrated excellent high‐rate performance, achieving discharge capacities of 122, 111, and 101 mAh g^−1^ at 3C, 5C, and 10C rates, respectively. The non‐toxic, solvent‐free synthesis of LATP‐NF, combined with its unique nanofibrous microstructure, underscores its great potential for advancing CPE development in solid‐state batteries.

## Conflict of Interest

The authors declare no conflict of interest.

## Supporting information



Supporting Information

## Data Availability

The data that support the findings of this study are available from the corresponding author upon reasonable request.
