# Supplementary material for: Microstructural Insights Into LATP Ceramic Nanofibers for High‐Performance Quasi‐Solid‐State Batteries
Source: Adv Sci (Weinh). 2025 Nov 20;13(7):e10846. doi: 10.1002/advs.202510846 (PMC12866698; doi:10.1002/advs.202510846)
Supplement: Supplementary file 1 — Supporting Information [file ADVS-13-e10846-s001.docx]

Supporting Information

Microstructural Insights into LATP Ceramic Nanofibers for High-Performance Quasi-Solid-State Batteries

Sivaraj Pazhaniswamy^1,2^*, Matteo Bianchini^3,2^, Shweta Hiwase^4,2^, and Seema Agarwal^4,2^*

^1^Department of Materials, University of Oxford, Parks Road, Oxford, OX1 3PH, United Kingdom,

^2^Bavarian Center of Battery Technology (BayBatt), Weiherstraße 26, 95448 Bayreuth, Germany

Email: [sivaraj.pazhaniswamy@materials.ox.ac.uk](mailto:sivaraj.pazhaniswamy@materials.ox.ac.uk)

^3^Faculty of Biology, Chemistry and Earth Sciences, University of Bayreuth, Universitätstraße 30, 95447 Bayreuth, Germany

^4^Advanced Sustainable Polymers, Macromolecular Chemistry II, University of Bayreuth, Bayreuth 95440, Germany

Email: agarwal@uni-bayreuth.de

**1. Experimental Section**

*Materials and Methods:* The following materials were used as raw materials for the preparation of LATP ceramic nanofibers: lithium acetate dihydrate (Li(C₂H₃O₂)·2H₂O, 98%, Sigma Aldrich), aluminum nitrate nonahydrate (Al(NO₃)₃·9H₂O, 99.995%, Sigma Aldrich), titanium isopropoxide (Ti[OCH(CH₃)₂]₄, 97%, Sigma Aldrich), ammonium dihydrogen phosphate (NH₄H₂PO₄, 99.99%, Sigma Aldrich), ammonium hydroxide (30% solution, Sigma Aldrich), poly(vinyl pyrrolidone) (PVP-1,300,000), phosphoric acid (H₃PO₄, 85%, Sigma Aldrich), and polyethylene oxide (PEO-600,000). For the preparation of solid polymer electrolytes, poly(vinylidene fluoride) (PVDF, MTI Corp.) and lithium bis(trifluoromethanesulfonyl)imide (LiTFSI, 99.95%, Sigma Aldrich) were used.

**1.1 Preparation of Electrospinning Solutions using PVP polymer**

*1.1.1 E-spinning solution-1 (****ES1****):* *Water + ethanol:* The stoichiometric amount of lithium acetate and aluminum nitrate were dissolved in ethanol (14 ml) stirred for 2 hours and the Titanium–isopropoxide was added into the above solution which was turned as white and stirred for 2 hours. Ammonium dihydrogen phosphate was dissolved in deionized water (6ml), and the resulting solution was added to the above mixture, which was then continuously stirred for 6 hours to obtain a white sol–gel precursor solution of LATP. The water and ethanol contents were 70:30% (v/v). The stoichiometric molar ratio of lithium (5% excess to compensate the loss of Li during calcination), aluminum, titanium and phosphorus was 1.4:0.4:1.6:3 for all the E-spinning solutions. The different wt% of PVP (5, 8, 12 and 16 wt%) was added into the LATP precursor solution as shown in **Figure S1a**. It was continuously stirred overnight to form the E-spinning solution of LATP and PVP named as ES1.

*1.1.2 E-spinning solution-2 (****ES2****): Water + ethanol + acetic acid****:*** In a typical procedure, lithium acetate and aluminum nitrate were dissolved in water and ethanol (5 ml+5ml), the Titanium–isopropoxide was dissolved in acetic acid (5ml) and the ammonium dihydrogen phosphate was added in water (5ml). All the solutions were continuously stirred for 4 hours separately then mixed together all and stirred for 6 hours to form a homogeneous sol-gel solution of LATP precursors (**Figure S1e**). The electrospinning solution was prepared by mixing two different concentration of (7.5wt% and 10 wt%) PVP into the LATP precursor solution. The ES2 solution was named as ES2a for 7.5wt% and ES2b for 10 wt% of PVP, respectively.

*1.1.3 E-spinning solution-3 (****ES3****): Water + ethanol* *+ isopropanol****:*** Lithium acetate and aluminum nitrate were added in ethanol and isopropanol (4ml+4ml) stirred for 2 hours and Titanium–isopropoxide was dissolved in the above solution and stirred for 2 hours. The ammonium dihydrogen phosphate was dissolved in water (3ml) separately 2 hours and mixed together all the solution and continuously stirred at least for 6 hours to form a LATP precursor solution. The ES3 was prepared by mixing two different concentrations (7.5 and 10 wt%) of PVP with the LATP precursor solution. The ES3 solution was named as ES3a for 7.5wt% and ES3b for 10 wt% of PVP, respectively.

***1.2 Preparation of electrospinning solution using PEO polymer:***

*1.2.1 E-spinning solution-4 (****ES4****): Water + ethanol + isopropanol:* In a typical synthesis, titanium isopropoxide (2.5 ml, (2.407g)) was added to ammonium hydroxide (5 ml) produced a white gelatinous precipitate. The precipitate was washed (6 times) with a large amount of deionized water to remove the excess base (reached Ph:7) and placed in 10 ml of deionized water + ethanol + isopropanol (0.75:1:1). The 1 M oxalic acid (1.838g in deionized water + ethanol (14ml+6ml), Mw of oxalic acid: 90.03) (Aldrich, 98%) was added to produce a clear transparent solution of H_2_[TiO(C_2_O_4_)_2_]. Then stoichiometric amount of lithium acetate (0.7697g) (5% excess) and aluminum nitrate (0.7695g) were added to the above solution and stirred for 1 hour. Then ammonium dihydrogen phosphate (1.77g) was added in to the solution and stirred at 80 °C for 4 hours, the clear transparent solution was turned as white as shown in **Figure S2 a-c**. Then ES4 was prepared by adding 4 wt% of PEO in the LATP solution and it was continuously stirred for one day. However, this solution was not provided nanofibers.

*1.2.2 E-spinning solution-5 (****ES5)****):*  The titanium isopropoxide (2.5 ml (2.407 g)) was added to the ammonium hydroxide (5 ml), forming a white gelatinous precipitate. This precipitate was washed 10 times with deionized water to remove excess base until a neutral pH (~7) was achieved. The precipitate was then dispersed in 10 ml of a water-ethanol-isopropanol mixture (6, 2, and 2 ml, respectively). Subsequently, the oxalic acid solution (0.5 M; 1.338 g in deionized water and ethanol (14 + 6 ml) was added to form a clear, transparent solution of H₂[TiO(C₂O₄)₂]. A stoichiometric amount of aluminum nitrate (0.7695 g) and 5% excess lithium acetate (0.7697 g) were added to the solution, followed by stirring for 1 hour. Phosphoric acid (1.77 g) was then introduced instead of ammonium dihydrogen phosphate (used in ES4). The resulting clear, homogeneous LATP precursor solution was stirred for 4 hours (Figure S2 e-g). To prepare the electrospinning solution, 4 wt% of PEO was added to the LATP precursor solution, and the mixture was stirred continuously for 24 hours and obtained the final solution.

*Preparation of LATP Ceramic Nanofibers:* The electrospinning process was performed using the ES1 to E5 solution under the following conditions: feed rate of 0.5 ml/h, 16 cm distance between the needle and the collector, applied voltage of +20 kV (positive) and -0.8 kV (negative), and a collector rotation speed of 60 rpm. Temperature and humidity were maintained at 25 °C and 38–41%, respectively. The as-spun LATP nanofiber mat was dried at 200 °C in a vacuum oven overnight to remove residual solvents. The dried mat was then calcined at various temperatures (600–900 °C) in an air atmosphere for 4 hours following a controlled heating program (Figure S2l) to remove organic components and produce crystalline LATP ceramic nanofibers. The LATP nanofibers were gently ground using a mortar to slightly shorten the long fibers before incorporation into the polymer matrix. Care was taken not to apply heavy pressure in order to preserve the fibrous morphology and prevent structural damage.

*Preparation of LATP Ceramic Nanofibers-Integrated Composite Polymer Electrolytes (CPE):* The solid polymer electrolyte (SPE) film was prepared by dissolving PVDF powder in DMF (16 wt%) with stirring at 60 °C until a clear and viscous solution formed. LiTFSI was added to the solution and stirred until homogeneous. The composition of PVDF and LiTFSI was 60 wt% and 40 wt%. The resulting PVDF-LiTFSI solution was cast onto a clean glass plate using a doctor blade to ensure uniform thickness. The solvent was evaporated at room temperature under a controlled atmosphere to minimize defects, and the film was dried in a vacuum oven at 100 °C to remove residual solvent, yielding flexible SPE film. For CPE films, the same procedure was followed, with the addition of LATP-NF. Appropriate amount of LATP-NF was dispersed in DMF via ultrasonication to ensure uniform dispersion before being added to the PVDF-LiTFSI solution. The mixture was stirred overnight and the resultant solution was treated by probe sonication (Branson 450 digital signifier) for 2 minutes, cast onto a glass plate using a doctor blade, and dried under the same conditions as SPE film. Also, CPE films were prepared with varying LATP-NF concentrations, as detailed in Table S2 and 4. The films were punched into 16 mm disks and stored in a glovebox (H₂ < 0.1 ppm, O₂ < 0.1 ppm) to prevent contamination or moisture absorption.

**1.3 Materials Characterization***:* Surface and cross-sectional microstructures were analyzed using field-emission scanning electron microscopy (FE-SEM, Zeiss Leo 1530). X-ray diffraction (XRD) analysis was performed using a Bragg-Brentano diffractometer (Cu K-α radiation, *λ* = 1.54187 Å, 2θ = 5–90°). Thermogravimetric analysis (TGA) was conducted from 25 to 1000 °C at a heating rate of 10 °C/min in air for LATP-NF and in N₂ for CPE and SPE films. Fourier transform infrared (FTIR) spectroscopy (PerkinElmer) was used to study the interactions between LATP-NF, polymers, and salts. Tensile strength and flexibility were measured using a universal tensile testing machine (Zwick/Roell, BT1-FR0.5TN. D14) with a tensile speed of 5 mm/min. Sample dimensions were 20 mm × 2 mm. Electrochemical impedance spectroscopy (EIS) was employed to measure the ionic conductivity of CPE and SPE. It was performed on 1.226 cm² films placed between stainless steel electrodes in a Swagelok cell. Measurements were taken at room temperature using a Gamry Reference 6000 system over a frequency range of 1 Hz to 2 MHz, with an applied voltage of 10 mV. The ionic conductivity of the CPE and SPE was calculated using the following equation:

*σ* = $\frac{t}{A{*R}_{b}}$ (1)

Here, σ represents the bulk ionic conductivity (S cm⁻¹), *t* is the thickness (cm) of the CPE and SPE, *A* is the electrolyte's area (cm²), and *R_b_* is the bulk resistance (Ω) (Table S3, S5 and S6). The bulk resistance was determined from the Nyquist plot by fitting the data using an equivalent circuit model. The electrochemical stability window of the CPE and SPE was determined by Linear Sweep Voltammetry (LSV, Gamry 6000) with potential range of 2.5 to 6 V at scan rate 0.25 mV s^-1^.

Lithium plating/stripping performance of CPE and SPE was examined by assembling Li|LATP-30|Li and Li|SPE|Li symmetric coin cells (Li size: 14 mm and CPE/SPE: 16 mm). The long-term lithium plating/stripping cycling performance of the cells was tested by charging/discharging at different constant current densities of 0.06, 0.1, and 0.5 mA cm⁻² for 1000, 1250 and 1500 cycles at 25°C. The cells were charged/discharged for 30 minutes during each cycle (Neware Battery Tester). The critical current density (CCD) of the Li|LATP-30|Li and Li|SPE|Li cells was determined by applying different current densities ranging from 0.01 to 10 mA cm⁻², with step increases of 0.01, 0.1, and 1 mA cm⁻². The 6 µl/cm^2^ liquid electrolyte (1 M LiPF₆ in EC:DMC = 1:1 vol%, with 5% FEC) was drop-cast on the lithium disk before assembling the symmetric cells to achieve good interface contact between lithium and solid electrolytes. Post-CCD tests, FE-SEM was performed on the CPE and SPE samples to evaluate their microstructural properties. Electrochemical impedance spectroscopy (EIS) was conducted before and after CCD testing, with measurements taken over a frequency range of 1 Hz to 100 kHz.

*Quasi-Solid-State Battery Assembly and Electrochemical Measurements:* The composite cathode electrode was fabricated using the slurry-casting technique. PVDF binder (7 wt%) was dissolved in NMP to form a homogeneous solution. The active material, LiFePO₄ (LFP) (MTI Co.) (70 wt%), LATP-NF (20 wt%), and carbon black (C65) (3 wt%) (MTI Co.) were added to the binder solution. The solution was stirred overnight using a magnetic stirrer to achieve a uniform slurry. The slurry was coated onto carbon-coated aluminum foil using an electrode coating machine. The coated foil was placed in a vacuum oven and dried at 120°C to remove the solvent completely. The dried electrode was roll-pressed at 50 N m^-2^ at 80°C to achieve a uniform electrode with an average mass loading of 2.11 mg cm^-2^. The same procedure and composition of active material, binder, and conductive carbon were employed to prepare the LiNi₀.₈Co₀.₁Mn₀.₁O₂ (NMC811) cathode (MSE Supplies, USA) with a mass loading of 3.5 mg cm⁻². A precise cutter was used to cut the dried electrode into disks (13 mm). The prepared cathode electrodes (disks) were stored in a glovebox (H₂ < 0.1 ppm and O₂ < 0.1 ppm) to prevent contamination or moisture absorption. The punched electrodes and solid electrolytes were dried in a vacuum oven at 80°C overnight before cell assembly. Coin-type QSLMBs (CR2032) were assembled in a glovebox with H₂/O₂ levels <0.1 ppm. A small amount of liquid electrolyte was introduced at both interfaces (Li|CPE/SPE and LFP|CPE/SPE) to enhance interfacial wetting and reduce interfacial resistance in the LFP‖CPE/SPE‖Li cells. Specifically, 6 μL cm⁻² of liquid electrolyte (1 M LiPF₆ in EC:DMC = 1:1 vol%, with 5% FEC) was carefully applied. The electrolyte was gently dropped and evenly spread onto the LFP cathode (13 mm diameter) using a fine, pointed hair brush before placing the best-performing CPE (LATP-30)/SPE film (16 mm) on top. An equal amount of liquid electrolyte was then applied to the surface of a well-polished lithium metal disk (14 mm), which was subsequently placed on the CPE/SPE–cathode assembly to fabricate the coin-type QSLMBs. The electrochemical performance of the LFP|CPE (LATP-30)|Li and LFP|SPE|Li SSB cells was examined using galvanostatic charge/discharge (GCD) tests (Neware Battery Tester). Cell formation was achieved by placing the cells at 25°C in a temperature-controlled chamber and charging/discharging them at a low current (0.1C) for 5 cycles. The cycling stability of the cells was studied at two different current rates (0.5C and 1C) (the theoretical capacity of LFP, 1C = 170 mAh/g) within a potential range of 2.5–4.2 V. The rate performance was examined by varying the current rates from 0.1C to 10C.


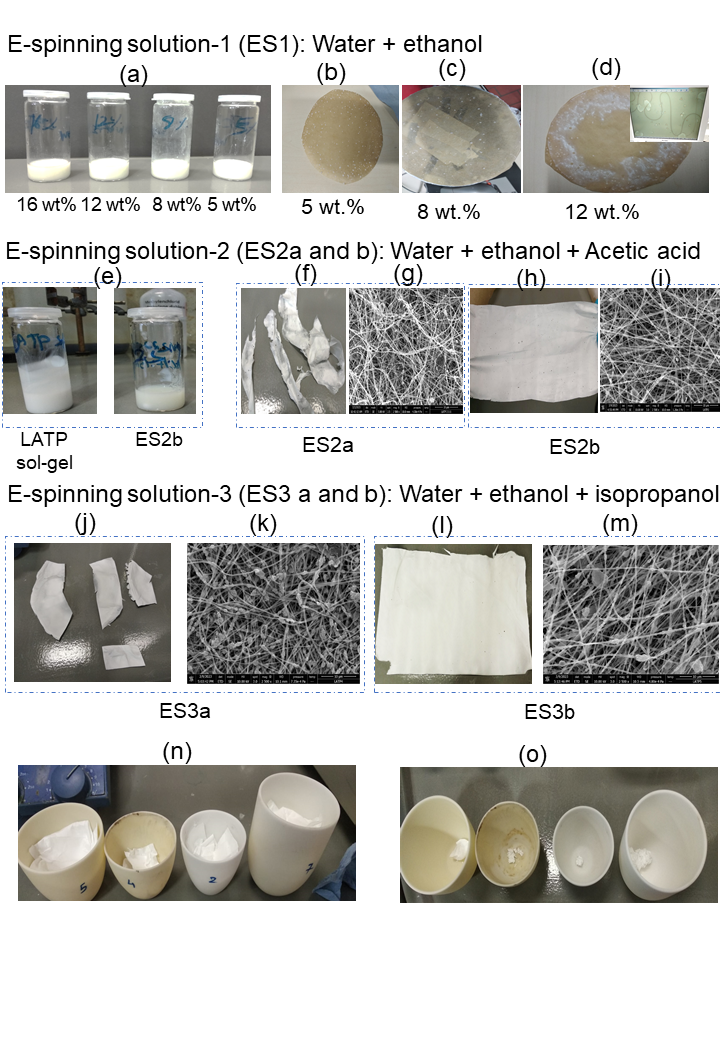


**Figure S1.** **ES1**: a) Photograph of **ES1** with different concentration of PVP, (b-d) electrospun mat with different concentration of PVP (only beads did not form nanofibers); **ES2 a&b**: (e) LATP precursor solution and ES2b solution, (f,g) photographs of electrospun mat and corresponding micrograph of LATP prepared by ES2a and (h,i) free standing mat and corresponding FE-SEM micrograph LATP prepared by ES2b; **ES3a&b**: (j,k) photographs of electrospun mat and corresponding FE-SEM micrograph of LATP prepared by ES3a and (l,m) free standing electrospun mat (large size) and FE-SEM micrograph of LATP prepared by ES3b; (n) electrospun mats before calcination and (o) LATP ceramic nanofibers after calcination


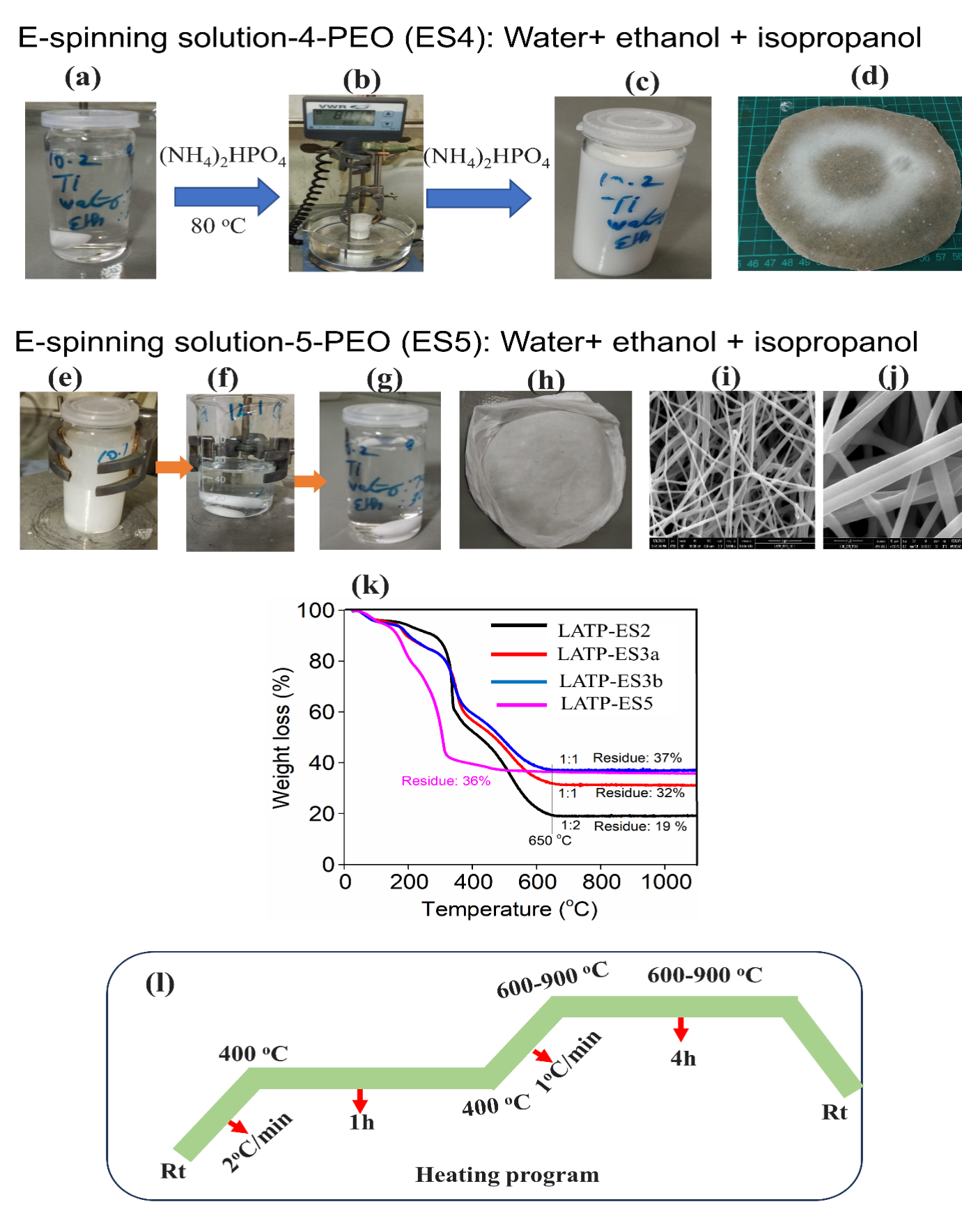


**Figure S2.** Preparation of LATP nanofibers using PEO polymer: **ES4**: (a) LATP precursor solution, (b) LATP-solution after adding (NH_4_)_2_HPO_4_), (c) LATP-sol-gel solution, (d) electrosupun mat (did not form the nanofibers); **ES5**: (e) Ti-precipitate in water + ethanol + isopropanol (after washing), (f) transparent Ti-solution, (g) transparent clear LATP solution, (h) electrospun LATP mat and (i and j) corresponding FE-SEM micrographs; (k) comparison of TGA curve of LATP-NF, and (l) specific heating program to obtain LATP-NF


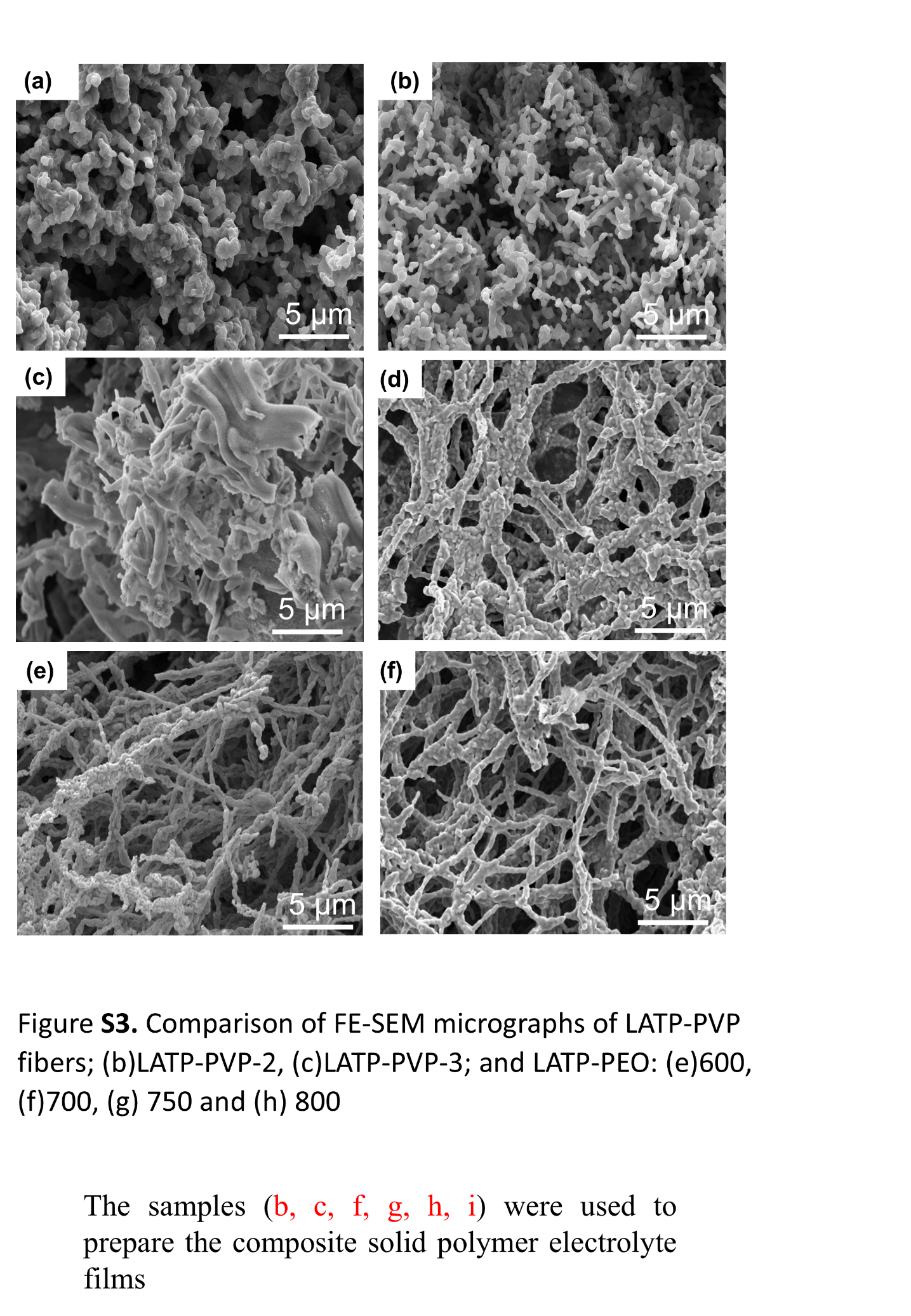


**Figure S3.** Selected LATP-NF morphologies for the fabrication of CPE films; FE-SEM micrographs: (a)ES3a, (b) ES3b, (c) ES5:600, (d) ES5:700, (e) ES5:750 and (f) ES5:800


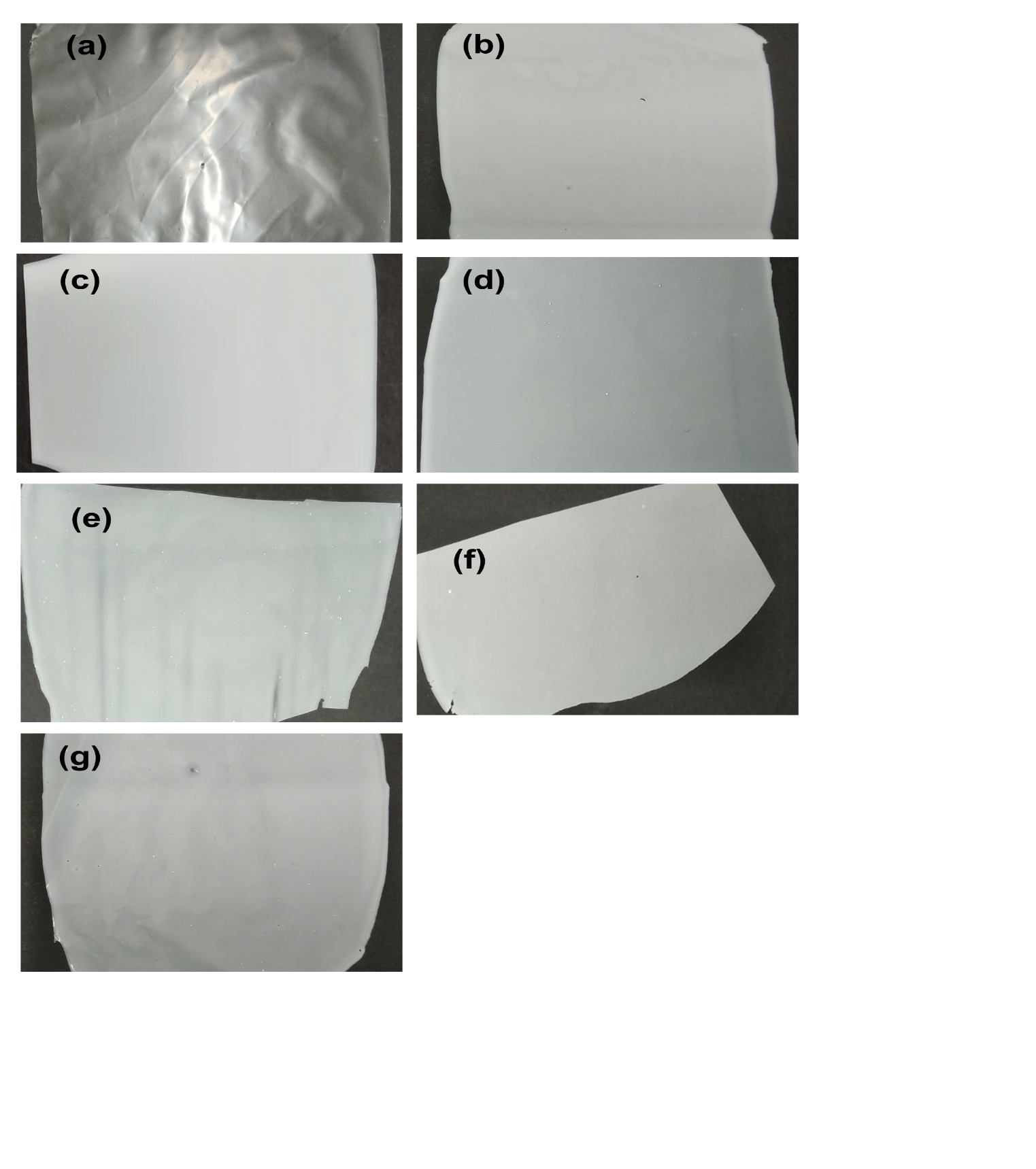


**Figure S4.** Photographs of SPE and CPE films: (a) SPE, (b) CPE-ES3a, (c) CPE-ES3b, (d) CPE-ES5-600, (e) CPE-ES5-700, (f) CPE-ES5-750 and (g) CPE-ES5-800 (All the films’ size ~10 cm x 8 cm)


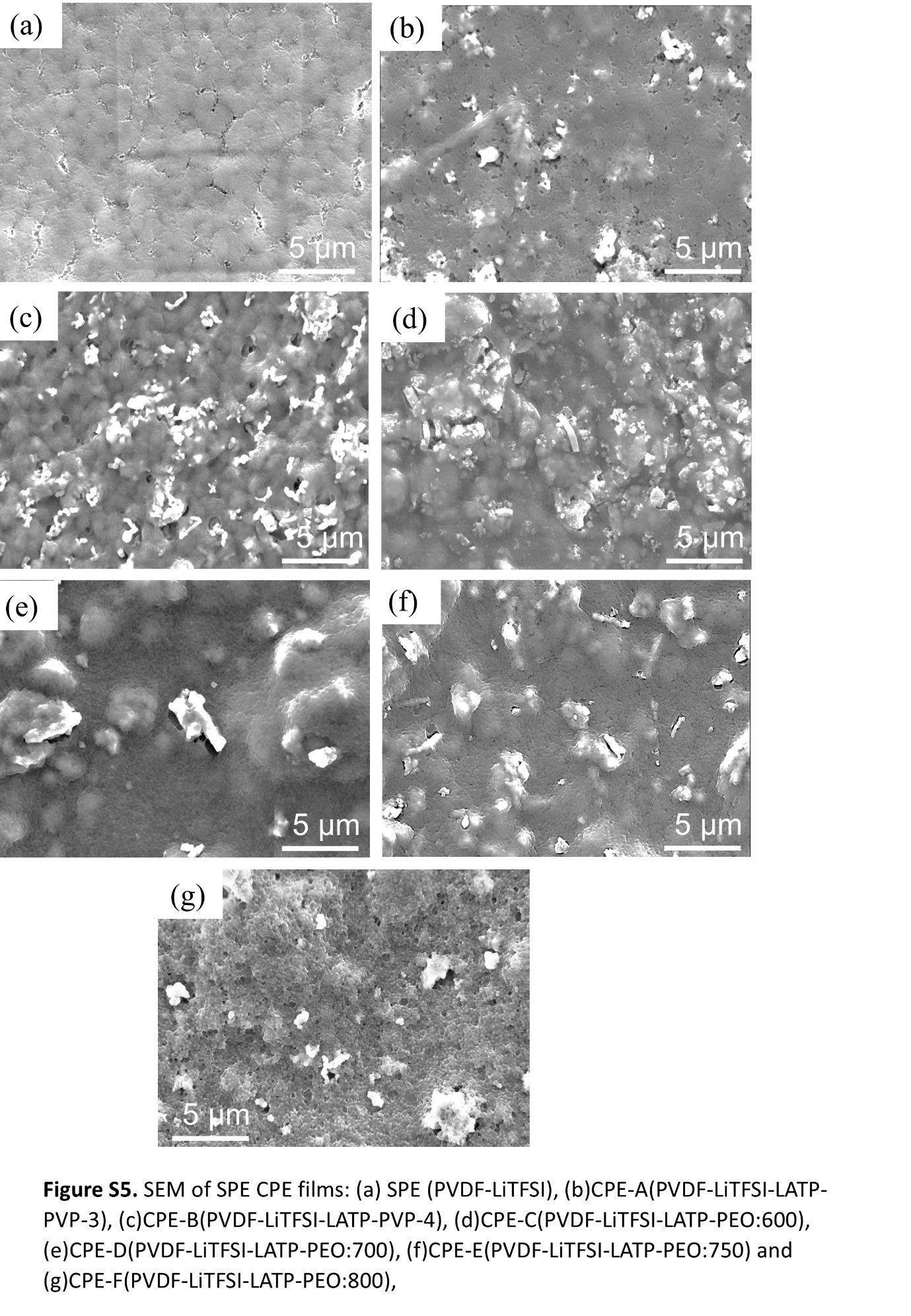


**Figure S5.** FE-SEM micrographs of SPE and CPE films: (a) SPE, (b) CPE-ES3a, (c) CPE-ES3b, (d) CPE-ES5-600, (e) CPE-ES5-700, (f) CPE-ES5-750 and (g) CPE-ES5-800


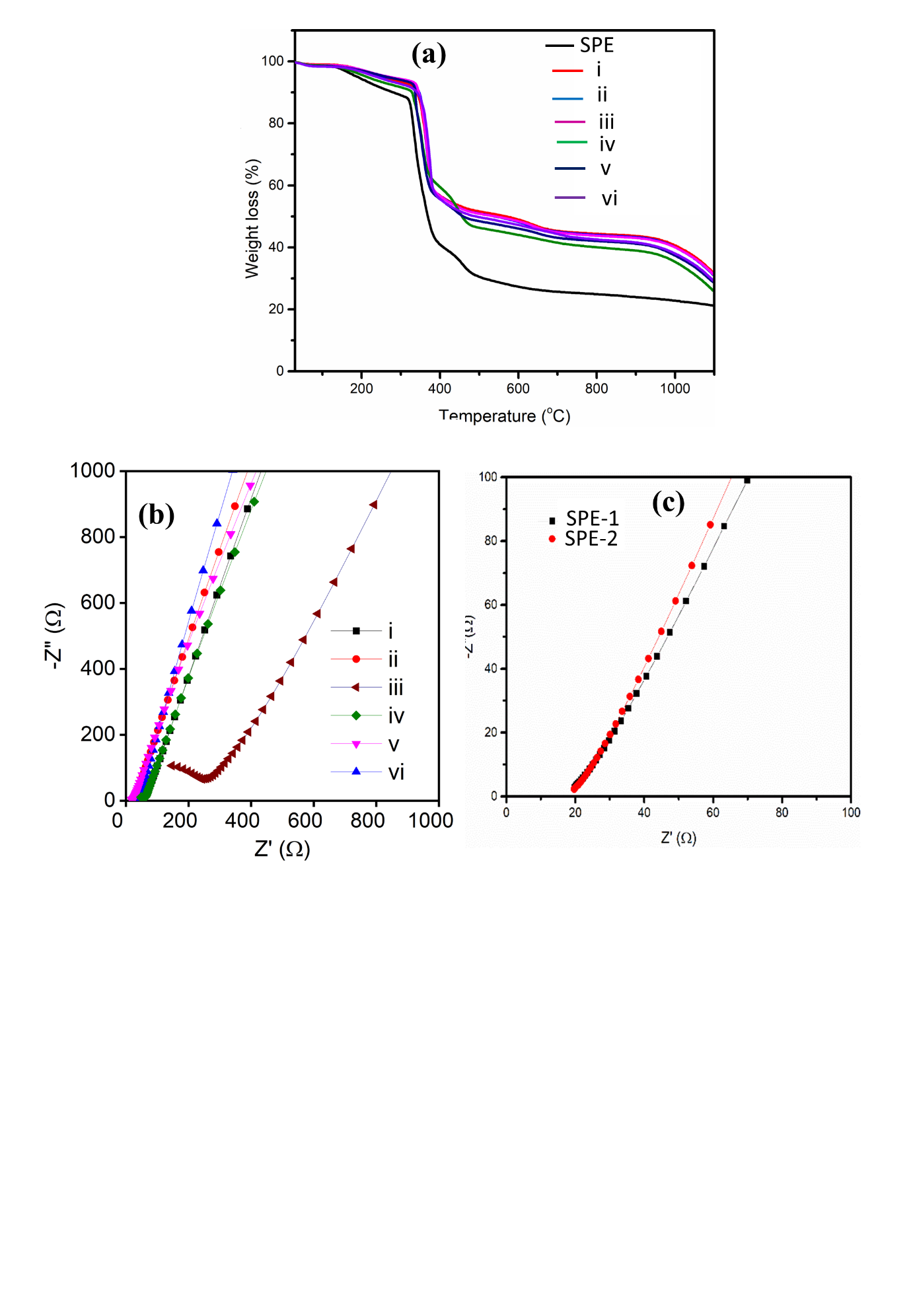


**Figure S6.** (a) TGA curves of SPE and CPE films, (b) Nyquist plots of CPE and (c) Nyquist plot of SPE (i: CPE-ES3a, ii: CPE-ES3b, iii: CPE-ES5:600, iv: CPE-ES5:700, v: CPE-ES5:750 and vi: CPE-ES5:800)


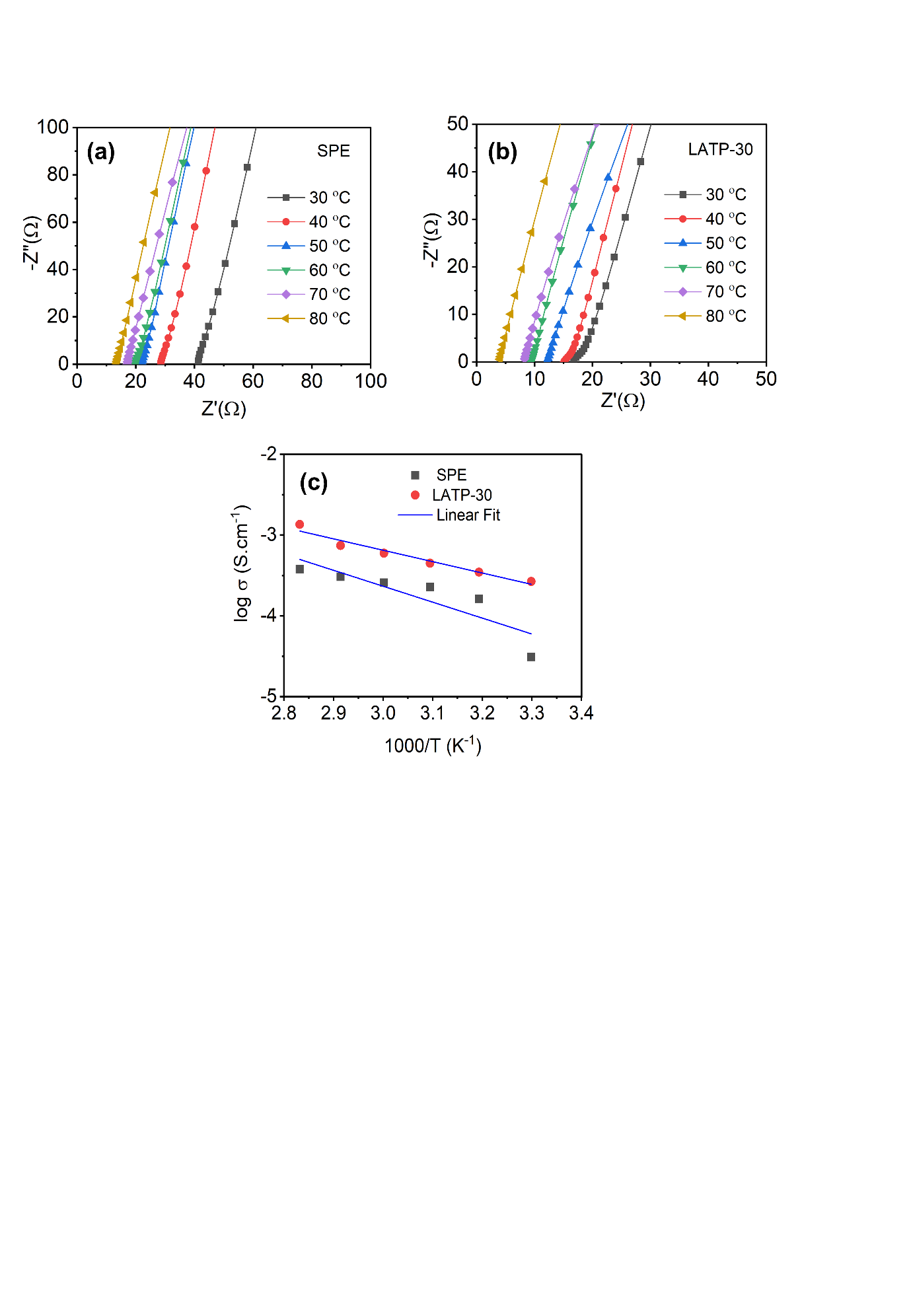


**Figure S7**. Temperature dependant conductivity; (a and b) Nyquist plots of SPE and LATP-30 with different temperatures (30-80 ^o^C) and (c) Arrhenius plot of SPE and LATP-30. (Area of SPE and LATP-30 films = 2.0096 cm^2^ (1.6 cm diameter) and Thickness of SPE =25 µm and LATP-30 =27 µm)


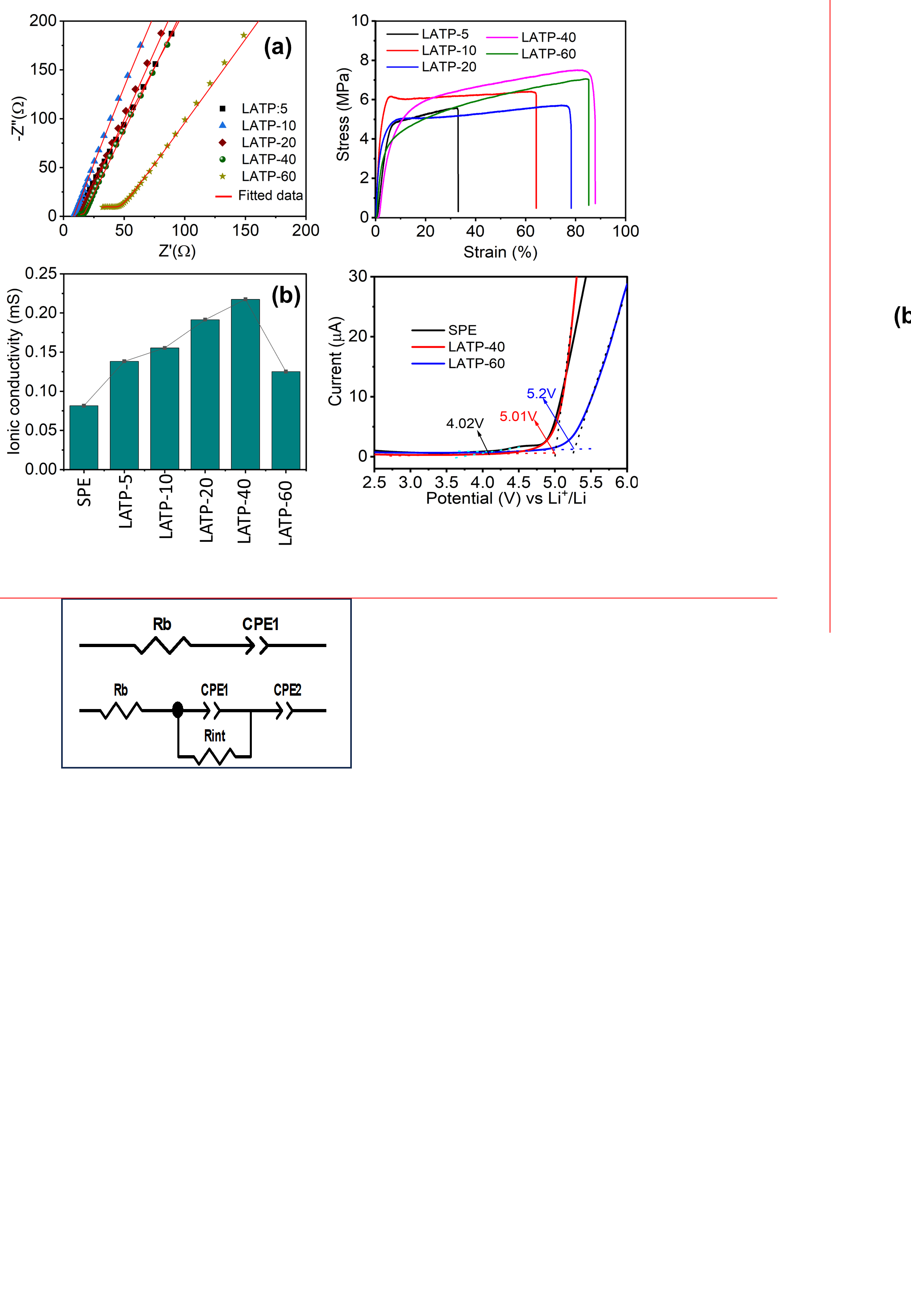


**Figure S8.** Most suitable equivalent circuit models


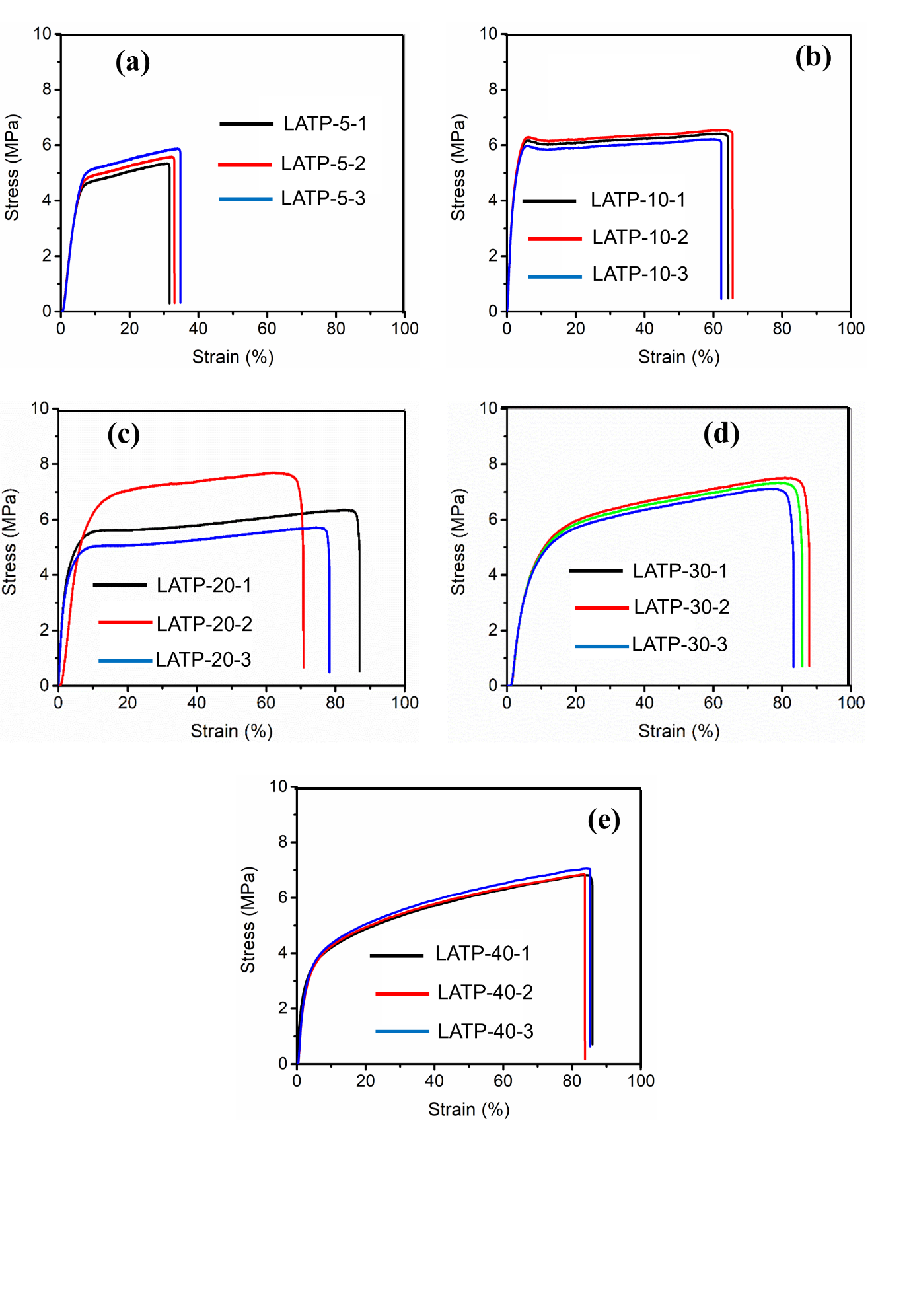


**Figure S9:** Stress vs Strain curves of CPE with different concentrations of ES5:750 ceramic nanofibers: (a) LATP-5, (b) LATP-10, (c) LATP-20, (d) LATP-30 and (e) LATP-40


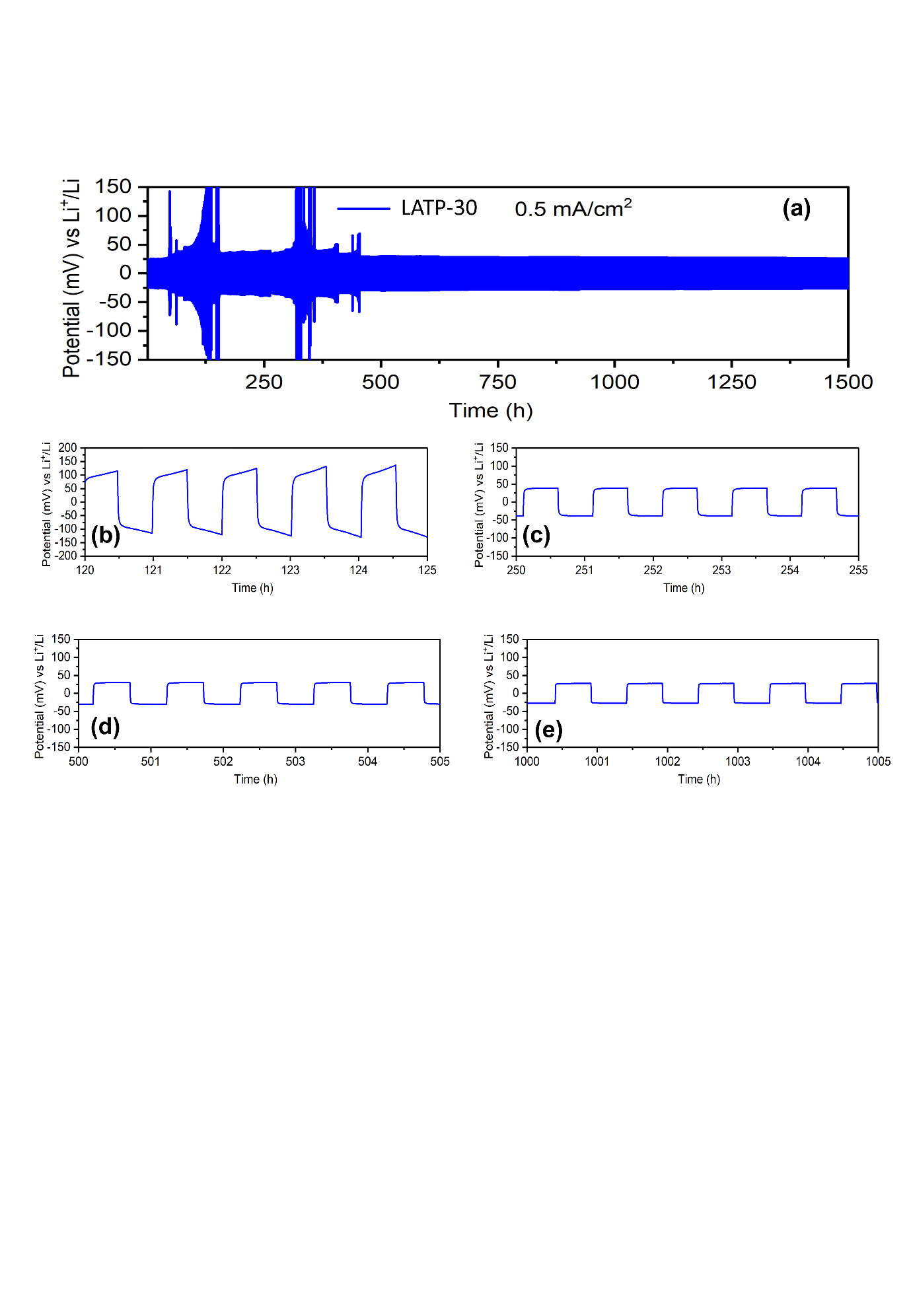


**Figure S10.** Long-term lithium platting/stripping cycling (GCD) of Li|LATP-30|Li cells: (a) at 0.5 mA/cm^2^ for 1500 cycles and (b-e) corresponding enlarged GCD curves


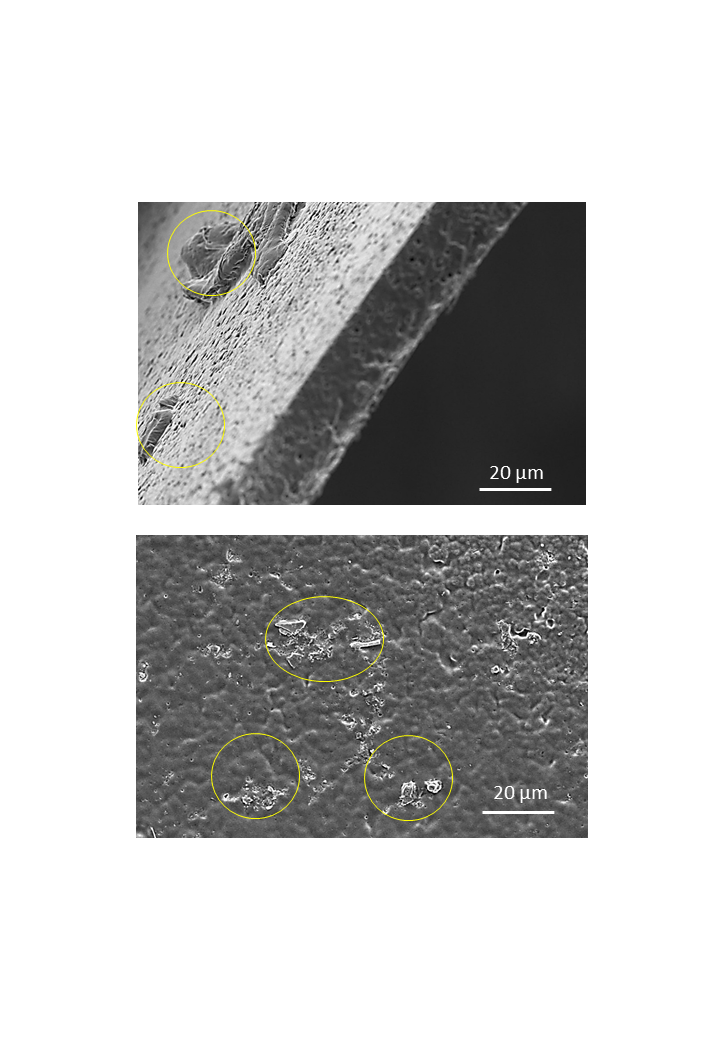


**Figure S11**: (a) Cross-sectional and (b) surface FE-SEM micrographs of SPE after CCD test


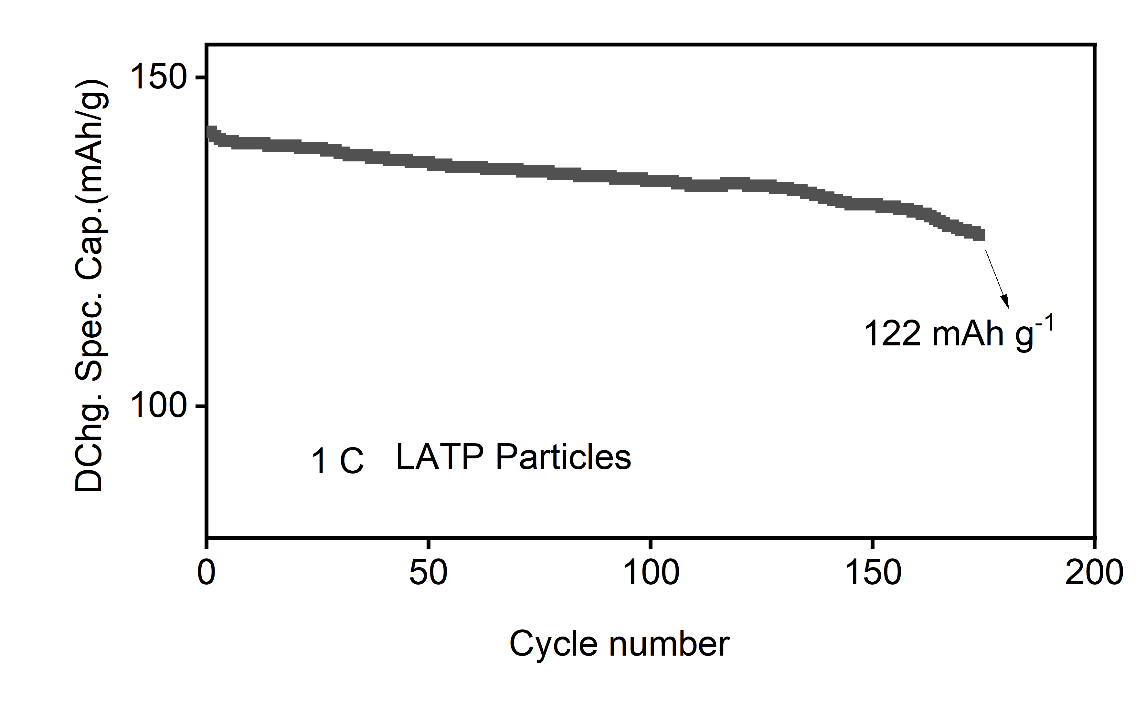


**Figure S12**: Long-term cycling stability of LATP particles (30 wt%) at 1 C rate


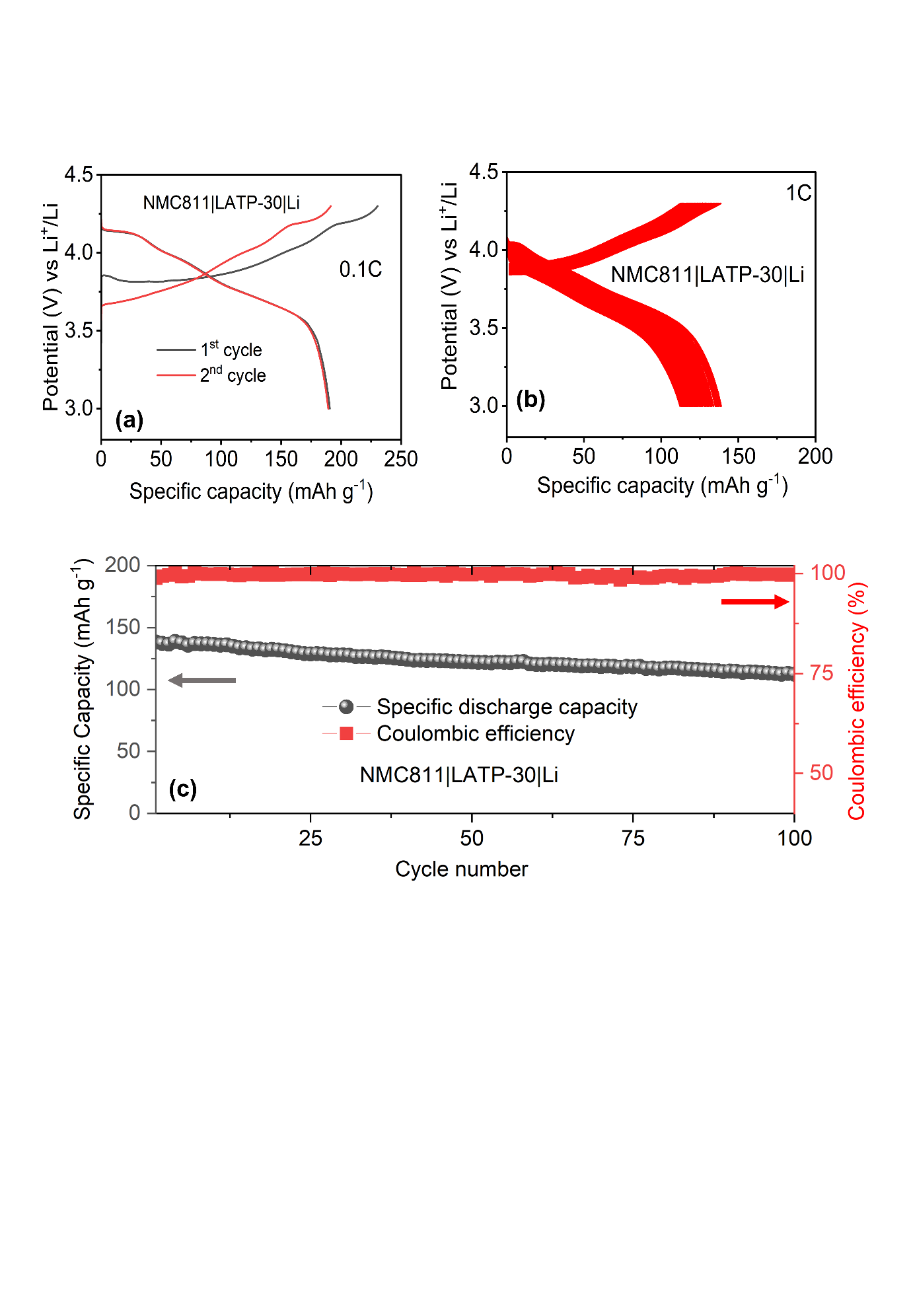


**Figure S13**: Electrochemical performance of NMC811|LATP-30|Li; (a and b) Potential vs specific capacity curves at 0.1 C and 1C, and (c) Long-term cycling stability at 1C

**Tables**

**Table S1.** Acronyms used in the manuscript

| Acronyms | Explanation |
| --- | --- |
| QSLMBs | Quasi-Solid-state lithium metal batteries |
| CPEs | Composite solid polymer electrolytes |
| LATP-NF | Li_1.4_Al_0.4_Ti_1.6_(PO_4_)_3_ ceramic nanofibers |
| CCD | Critical current density |
| LFP | LiFePO_4_ |
| SSEs | Solid-state electrolytes |
| PVDF | Polyvinylidene fluoride |
| LiTFSI | Lithium bis(trifluoromethane)sulfonimide |
| PVP | Poly(vinyl pyrrolidone) |
| PEO | Polyethylene oxide |
| ES | Electrospinning solutions |
| ES1 | Electrospinning solutions-1: Water + ethanol |
| ES2a | Electrospinning solutions-2a: Water + ethanol + acetic acid with 7.5wt% of PVP |
| ES2b | Electrospinning solutions-2b: Water + ethanol + acetic acid with 10wt% of PVP |
| ES3a | Electrospinning solutions-3a: Water + ethanol + isopropanol with 7.5wt% of PVP |
| ES3b | Electrospinning solutions-3b: Water + ethanol + isopropanol with 10 wt% of PVP |
| ES4 | Electrospinning solutions-4: Water + ethanol+ isopropanol with PEO polymer and ammonium dihydrogen phosphate used in LATP precursor |
| ES5 | Electrospinning solutions-5: A transparent ES solution contains Water + ethanol+ isopropanol with PEO polymer and phosphoric acid used in LATP precursor |
| ES5:600 | LATP-NF prepared by ES5 and calcined at 600 °C |
| ES5:700 | LATP-NF prepared by ES5 and calcined at 700 °C |
| ES5:750 | LATP-NF prepared by ES5 and calcined at 750 °C |
| ES5:800 | LATP-NF prepared by ES5 and calcined at 800 °C |
| ES5:900 | LATP-NF prepared by ES5 and calcined at 900 °C |
| CPE-ES3a | Composite polymer electrolyte prepared using LATP-NF synthesized by ES3a |
| CPE-ES3b | Composite polymer electrolyte prepared using LATP-NF synthesized by ES3b |
| CPE-ES5-600 | Composite polymer electrolyte prepared using LATP-NF synthesized by ES5 at 600 °C |
| CPE-ES5-700 | Composite polymer electrolyte prepared using LATP-NF synthesized by ES5 at 700 °C |
| CPE-ES5-750 | Composite polymer electrolyte prepared using LATP-NF synthesized by ES5 at 750 °C |
| CPE-ES5-800 | Composite polymer electrolyte prepared using LATP-NF synthesized by ES5 at 800 °C |
| LATP-5 | Composite polymer electrolyte prepared using 5wt% of LATP-NF synthesized by ES5 at 750 °C |
| LATP-10 | Composite polymer electrolyte prepared using 10 wt% of LATP-NF synthesized by ES5 at 750 °C |
| LATP-20 | Composite polymer electrolyte prepared using 20 wt% of LATP-NF synthesized by ES5 at 750 °C |
| LATP-30 | Composite polymer electrolyte prepared using 30 wt% of LATP-NF synthesized by ES5 at 750 °C |
| LATP-40 | Composite polymer electrolyte prepared using 40 wt% of LATP-NF synthesized by ES5 at 750 °C |

**Table S2.** The selected LATP-NF for the fabrication of CPE films and sample designation

| Sample designation | LATP-NF for CPE |
| --- | --- |
|  |  |
| SPE (PVDF-LiTFSI) | - |
| CPE-ES3a | ES3a |
| CPE-ES3b | ES3b |
| CPE-ES5-600 | ES5:600 |
| CPE-ES5-700 | ES5:700 |
| CPE-ES5-750 | ES5:750 |
| CPE-ES5-800 | ES5:800 |

**Table S3**. Bulk resistance and ionic conductivity of SPE and CPE films with LATP-NF prepared by different electrospinning solutions

| Samples | Bulk resistance (Ω) | Area (cm^2^) | Thickness (cm) | Conductivity (S cm^-1^) |
| --- | --- | --- | --- | --- |
| CPE-ES3a | 55 | 1.226 | 0.0026 | 3.86 x 10^-5^ |
| CPE-ES3b | 23 | 1.226 | 0.0027 | 9.58 x 10^-5^ |
| CPE-ES5:600 | 252 | 1.226 | 0.0025 | 8.09 x 10^-6^ |
| CPE-ES5:700 | 59 | 1.226 | 0.0029 | 4.01 x 10^-5^ |
| CPE-ES5:750 | 19 | 1.226 | 0.0037 | 1.60 x 10^-4^ |
| CPE-ES5:800 | 36 | 1.226 | 0.0026 | 5.89 x 10^-5^ |
| SPE | 20 | 1.226 | 0.0020 | 8.16 x 10^-5^ |

**TableS4.** Composition of LATP-NF (LATP-ES5:750), PVDF and LiTFSI for the fabrication of CPE with different concentration of ceramic nanofibers. Composition of PVDF-LiTFSI was fixed as 60:40 wt%

| CPE designation | Composition (wt%) | | Total weight of CPE (g) |
| --- | --- | --- | --- |
|  | PVDF: LiTFSI | ES5:750 |  |
| LATP-5 | 95 | 5 | 0.6 |
| LATP-10 | 90 | 10 | 0.6 |
| LATP-20 | 80 | 20 | 0.6 |
| LATP-30 | 70 | 30 | 0.6 |
| LATP-40 | 60 | 40 | 0.6 |

**Table S5.** Parameters of EIS data fitting with equivalent circuit models

| Sampe ID | Bulk resistance R_b_ (Ω) | CPE1  F.s^^(n-1)^ | n1 | R_int_ interfacial resistance (Ω) | CPE2  F.s^^(n-1)^ | n2 | χ^2^/\|Z\| |
| --- | --- | --- | --- | --- | --- | --- | --- |
| LATP-5 | 9.69 | 20.83 x 10^-6^ | 0.7425 | - | - | - | 0.014 |
| LATP-10 | 8.5 | 16.97 x 10^-6^ | 0.7753 | - | - | - | 0.093 |
| LATP-20 | 10.22 | 0.371x 10^-3^ | 0.3364 | 1.07 | 11.39 x 10^-6^ | 0.779 9 | 0.019 |
| LATP-30 | 12.22 | 47.96 x 10^-9^ | 0.99 | 2.6 | 11.83 x10^-6^ | 0.740 1 | 0.076 |
| LATP-40 | 21.5 | 0.301 x 10^-6^ | 0.729 | 24.66 | 12.17x10^-6^ | 0.663 6 | 0.0121 |

**Table S6.** Bulk resistance obtained by fitting the EIS data with equivalent circuit models and ionic conductivity of SPE and CPE with different concentration of ES5:750 ceramic nanofibers

| Samples | Bulk resistance (Ω) | Area (cm^2^) | Thickness (µm) | Conductivity (S cm^-1^) |
| --- | --- | --- | --- | --- |
| SPE | 20 | 1.226 | 20 | 8.16 x 10^-5^ |
| LATP-5 | 9.69 | 1.226 | 19 | 1.38 x 10^-4^ |
| LATP-10 | 8.5 | 1.226 | 20 | 1.55 x 10^-4^ |
| LATP-20 | 10.22 | 1.226 | 24 | 1.92 x 10^-4^ |
| LATP-30 | 12.22 | 1.226 | 32 | 2.18 x 10^-4^ |
| LATP-40 | 21.5 | 1.226 | 33 | 1.25 x 10^-4^ |

**Table S7.** Temperature dependant conductivity of the SPE and LATP-30 samples, area of SPE and LATP-30 films are 2.0096 cm^2^ (1.6 cm diameter) and Thickness of SPE =25 µm and LATP-30 =27 µm (Thickness and area were slightly changed for this film in order to validate reproducibility)

| Temperature (^o^C) | SPE  Total resistance (Ω) | LATP-30  Total resistance (Ω) | SPE  Total conductivity (S cm^-1^) | LATP-30  Total conductivity (S cm^-1^) |
| --- | --- | --- | --- | --- |
| 30 | 40.1 | 17.83 | 3.10 x 10^-5^ | 3.04 x 10^-4^ |
| 40 | 31 | 15.6 | 1.63 x 10^-4^ | 3.48 x 10^-4^ |
| 50 | 22 | 12.08 | 2.28 x 10^-4^ | 4.49 x 10^-4^ |
| 60 | 18 | 9.07 | 2.79 x 10^-4^ | 5.98 x 10^-4^ |
| 70 | 16.45 | 7.3 | 3.05 x 10^-4^ | 7.43 x 10^-4^ |
| 80 | 13.2 | 4.0 | 3.80 x 10^-4^ | 1.36 x 10^-3^ |

**Table S8.** Comparison of Lithium stripping plating performance including CCD /max current density used for dendrite study for the LATP particles/fibers with recent literature reports (2021-2025)

| Ref. number | LATP particle/fiber | Polymer/salt | Striping/plating current density mA/cm^2^ | Number cycles (h)/temperature (°C) | CCD values or maximum current density used (mA/cm^2^) | Liquid electrolyte or liquid additives ( μL) | Year of publication |
| --- | --- | --- | --- | --- | --- | --- | --- |
| This work | **LATP-NF** | PVDF-LiTFSI | 0.1/0.5 | 1200/25 | **10** | **6** μL /cm^2^ | **-** |
| 34 | LATP | PVDF-LiClO_4_ | 0.2 | 300/ Rt | **2** | 5 | 2025 |
| 35 | LATP | PVDF–LiTFSI | 0.1 | 300/60 | **0.2** | Immersed in LE | 2024 |
| 36 | LATP | *CLA-CN | 0.1 | 1200/ Rt | **0.64** | 50 | 2024 |
| 37 | LATP | *Pyr_14_TFSI-LiTFSI | 0.1 | 120/- | **0.1** | - | 2024 |
| 26 | LATP | PVDF-HFP-SN-LiTFSI | 0.2 | 550/ Rt | **3** | 10 | 2024 |
| 38 | LATP | PVDF-HFP | 0.1 | 2000/ Rt | **1.3** | 60 | 2024 |
| 39 | LATP | PVDF-HFP | 0.2 | 200/Rt | **0.2** | 30 | 2023 |
| 40 | LATP | PVDF-*PVC-LITFSI | 0.2 | 600/25 | **0.2** | 40 | 2022 |
| 41 | LATP | PVDF-HFP-LITFSI | 0.5 | 500/25 | **1** | - | 2022 |
| 42 | LATP | Mg_3_N_2_-PVDF-LiTFSI | 0.2 | 1000/60 | **0.76** | 5 | 2022 |
| 43 | LATP | PVDF-SN-LiTFSI | 0.2/0.5 | ~100/25 | **0.5** | 9.1 | 2022 |
| 44 | LATP | PVDF-HFP | - | - | **1** | - | 2021 |
| 45 | LATP | PVDF-*EMI-, LiTFSI-*FEC | 0.1 | 600/25 | **0.7** | Immersed in *LE | 2021 |

CLA-CN: cellulose acetate quasi-solid composite electrolyte, *Pyr14TFSI: 1-butyl-1-methylpyrrolidinium bis (trifluoromethanesulfonyl)imide, *LABTP:Li1.3Al0.3-xBixTi1.7(PO4)3 (x = 0, 0.01, 0.02, 0.03), EMI:1-ethyl-3-methylimidazolium bis-(triﬂuoromethanesulfonyl)imide, FEC: Fuoroethylene carbonate, *LE: Liquid electrolytes,

**Table S9.** Comparison of the electrochemical performance of LATP based CPE with LATP particles/fibers including maximum rate capacity (recent literature reports: 2021-2025)

*Spc.Cap: Specific capacity,

| Ref. number | LATP particle/fibers | Polymer-salt | Cathode | *Spc.Cap. after cycling (mAh/g) | No. of cycles / temperature (°C) | Capacity retention (%)/C-rate | Spc.Cap. at maximum C rate (mAh/g) | Highest C rate | Liquid electrolyte or liquid additives ( μL) | Year of publication |
| --- | --- | --- | --- | --- | --- | --- | --- | --- | --- | --- |
| This work | **LATP-NF** | **PVDF-LiTFSI** | **LFP** | **152** | **300/25** | **97/0.5** | **111 & 101** | **5 & 10** | **6** μL /cm^2^ | **-** |
| 34 | LATP | PVDF-LiClO_4_ | LFP | 105 | 100/ | -/0.2 | 127 | **1** | 5 | 2025 |
| 35 | LATP | PVDF–LiTFSI | LFP | - | - | - | 100 | **1** | Immersed in LE | 2024 |
| 36 | LATP | *CLA-CN | LFP | - | 1500/25 | 92.7/0.5 | 117.3 | **1** | 50 | 2024 |
| 37 | LATP | *Pyr_14_TFSI-LiTFSI | LFP | - | - | - | 60 | **1** | - | 2024 |
| 26 | LATP | PVDF-HFP-SN-LiTFSI | LFP | 119 | 200/RT | 85.5/0.5 | ~130 | **2** | 10 | 2024 |
| 38 | LATP | PVDF-HFP | LFP | 122 | 300/RT | 95.4/2 | 102 | **4** | 60 | 2024 |
| 39 | LATP | PVDF-HFP | LFP | - | 200/30 | - | 130 | **1** | 30 | 2023 |
| 40 | LATP | PVDF-*PVC-ITFSI | LFP | - | 200/25 | 94.1/1 | 124.5 | **2** | 40 | 2022 |
| 41 | LATP | PVDF-HFP-LITFSI | LFP | 143 | 600/- | 94.7/1 | ~85 | **3** | - | 2022 |
| 42 | 124.5LATP | Mg_3_N_2_-PVDF-LiTFSI | LFP | - | 417/60 | 80/1 | 144.2 | **4** | 5 | 2022 |
| 43 | LATP | PVDF-SN-LiTFSI | LFP | - | 250/25 | 96/0.1 | 13.55 | **4** | 9.1 | 2022 |
| 44 | LATP | PVDF-HFP | LFP | - | - | - | 146.6 | **2** | - | 2021 |
| 45 | LATP | PVDF-*EMI-LiTFSI-*FEC | LFP | - | 200/25 | 96.5/0.5 | 75.66 | **2** | Immersed in LE | 2021 |
